# A non-destructive sugar-feeding assay for parasite detection and estimating the extrinsic incubation period of *Plasmodium falciparum* in individual mosquito vectors

**DOI:** 10.1038/s41598-021-88659-w

**Published:** 2021-04-29

**Authors:** Edwige Guissou, Jessica L. Waite, Matthew Jones, Andrew S. Bell, Eunho Suh, Koudraogo B. Yameogo, Nicaise Djègbè, Dari F. Da, Domonbabele F. D. S. Hien, Rakiswende S. Yerbanga, Anicet G. Ouedraogo, Kounbobr Roch. Dabiré, Anna Cohuet, Matthew B. Thomas, Thierry Lefèvre

**Affiliations:** 1 Institut de Recherche en Sciences de la Santé, Bobo-Dioulasso, Burkina Faso; 2 MIVEGEC, Montpellier University, IRD, CNRS, Montpellier, France; 3 Laboratoire mixte international sur les vecteurs (LAMIVECT), Bobo Dioulasso, Burkina Faso; 4 Université Nazi Boni, Bobo Dioulasso, Burkina Faso; 5 Center for Infectious Disease Dynamics, The Pennsylvania State University, University Park, PA 16802 USA; 6 Green Mountain Antibodies, Inc. 1 Mill St. Suites 1-7, Burlington, VT 05401 USA; 7 York Environmental Sustainability Institute and Department of Biology, University of York, York, UK; 8 Centre de Recherche en Écologie et Évolution de la Santé (CREES), Montpellier, France

**Keywords:** Ecology, Evolution, Molecular biology, Zoology, Medical research, Diseases, Infectious diseases

## Abstract

Despite its epidemiological importance, the time *Plasmodium* parasites take to achieve development in the vector mosquito (the extrinsic incubation period, EIP) remains poorly characterized. A novel non-destructive assay designed to estimate EIP in single mosquitoes, and more broadly to study *Plasmodium*–*Anopheles* vectors interactions, is presented. The assay uses small pieces of cotton wool soaked in sugar solution to collect malaria sporozoites from individual mosquitoes during sugar feeding to monitor infection status over time. This technique has been tested across four natural malaria mosquito species of Africa and Asia, infected with *Plasmodium falciparum* (six field isolates from gametocyte-infected patients in Burkina Faso and the NF54 strain) and across a range of temperatures relevant to malaria transmission in field conditions. Monitoring individual infectious mosquitoes was feasible. The estimated median EIP of *P. falciparum* at 27 °C was 11 to 14 days depending on mosquito species and parasite isolate. Long-term individual tracking revealed that sporozoites transfer onto cotton wool can occur at least until day 40 post-infection. Short individual EIP were associated with short mosquito lifespan. Correlations between mosquito/parasite traits often reveal trade-offs and constraints and have important implications for understanding the evolution of parasite transmission strategies.

## Introduction

The extrinsic incubation period (EIP) of *Plasmodium* is the duration of the parasite’s development within the mosquito that starts with the ingestion of gametocytes (the human to mosquito transmission stage) in a blood meal and ends with the sporozoites invasion of the salivary glands when the mosquito becomes infectious^1,2^. Following the consumption of gametocyte-positive blood, the male and female gametocytes first egress from the enveloping erythrocytes and fertilize in the mosquito midgut. Within a few hours, the resulting zygotes complete a meiosis and develop into motile ookinetes that cross the midgut wall and lodge to the basal lamina of the midgut where they mature into oocysts. Within the oocysts, mitotic divisions produce large number of sporozoites, which break out into the mosquito body cavity, and invade the mosquito salivary glands at least 1 week post-infection (depending on the malaria species^3–5^ and temperature^2,6–9^. EIP plays a crucial role in malaria vectorial capacity (a standard measure of malaria transmission potential), as small changes in EIP can have a large effect on the number of mosquitoes living long enough to be able to transmit parasites^10–12^. The earlier the parasite invades mosquito salivary glands, the sooner the vector becomes infectious, and will have the potential to infect more humans over its lifespan.

Despite such epidemiological importance, the estimation of EIP has been limited to a small number of vector-parasite combinations, and sources of variability in EIP remain largely unknown. Besides temperature and parasite species, evidence that EIP can be influenced by other genetic and environmental factors, and possibly be associated with other key transmission parameters such as mosquito longevity, is limited^1,2^. The reason for this gap of knowledge probably lies in the difficulty of measuring EIP in the laboratory. Ohm et al.^2^ showed that EIP_50_ (the time until 50% of infected mosquitoes become infectious, which equates to the median time of sporozoites development) is the most useful measure for transmission modeling, but finding the midpoint of a transmission curve is labor intensive. Current methods consist in experimentally infecting a large batch of mosquitoes and dissecting a number of salivary glands at several time points (e.g. every day or two) to monitor parasite maturation. This process suffers several limitations. First, it requires infecting several hundreds of mosquitoes at the same time. This number increases if there is not an already well-defined time range for the EIP of the studied parasite species, or when infection prevalence is low. Second, it requires skilled researchers to dissect mosquito salivary glands and examine them for sporozoites under high magnification (400–1000×). Third, this approach results in an estimation of EIP at the mosquito population level, hence preventing the characterization of direct associations between EIP and other life-history traits such as mosquito longevity, fecundity, or parasite density. Faced with these limitations, other researchers have tried to solve the problems by developing new methods. These methods consist in marking parasites with a green fluorescent protein (GFP) to allow parasites to be seen without necessarily dissecting the mosquitoes. However, this also limits the scope of the work of using experimentally infected mosquitoes with these GFP strains only, and is still technically complex^13,14^. As an alternative to mosquito dissection, Billingsley et al.^15^ developed an antibody based blot assay to see if sporozoites were deposited with mosquito saliva on a sugar substrate, their successes suggesting this technique could be further developed.

In the context of large-scale vector-borne disease surveillance and elimination programs, some systems have been developed whereby the sugar feeding behavior of mosquito vectors is similarly exploited to detect pathogens. As during blood feeding, mosquito vectors salivate during sugar feeding, not only to facilitate ingestion by diluting concentrated nectars, but also to initiate sugar digestion^16^. While salivating, pathogens can be expelled. New field surveillance systems rely on traps where mosquitoes are provided access to sugar-baited substrates from which expelled pathogens can then be detected using qRT-PCR. Such sugar-based surveillance systems to track pathogens in wild mosquito populations have now been successfully applied to arboviruses^17–24^ and are currently being developed for malaria^25–27^.

Pathogen detection through mosquito sugar feeding has also recently been used in laboratory settings for investigating mosquito-virus interactions. Applications include the potential use of *Wolbachia* to reduce dengue transmission^28^, the effect of *Aedes aegypti* genetic variation on the EIP of a dengue virus^29^, the temporal dynamic of mosquito competence for dengue and Chikungunya viruses^30,31^, the changes in viral population structure over time^32^, mosquito immune response to Chikungunya and Zika viruses^33^ and characterizing the EIP of a West Nile Virus in *Culex tarsalis*^34^. Repeated sampling using this non-destructive method requires fewer mosquitoes and offers a unique opportunity to investigate the dynamic nature of vector-pathogen interactions.

In this study, a novel non-destructive assay is described based on the exploitation of mosquito sugar feeding to reveal infection with malaria parasites and designed to study *Plasmodium*–*Anopheles* vectors interactions. In particular, we provide proof-of-concept that this assay can be used to estimate *Plasmodium* EIP in single mosquitoes across a range of different vector species, parasite strains and densities, and temperatures.

## Methods

### Mosquito colonies and maintenance

Experiments were conducted between two labs, the one at the Institut de Recherche en Sciences de la Santé (IRSS, Bobo Dioulasso, Burkina Faso), and the other at Pennsylvania State University (PSU, Pennsylvania, USA), and used five mosquito colonies in total.

1. *Anopheles gambiae* (IRSS)*,* obtained from an outbred colony brought into the lab in 2008 and repeatedly replenished with F1 from wild-caught mosquito females collected in Soumousso, (11°23′14″N, 4°24′42″W), 40 km from Bobo Dioulasso, and identified by routine SINE PCR^35^.
2. *Anopheles coluzzii* (IRSS), obtained from an outbred colony brought into the lab in 2016 from wild-caught mosquito females collected in Kou Valley (11°24′N, 4°24′59″W), 30 km from Bobo Dioulasso, and identified by SINE PCR^35^.
3. *Anopheles arabiensis* (IRSS) obtained from an outbred colony brought into the lab in 2015 from wild-caught gravid females collected in Soumousso, and identified by routine SINE PCR^35^.
4. *Anopheles stephensi* (PSU) established in the lab from eggs shipped from Walter Reed (USA) in June 2015.
5. *An. gambiae/An. coluzzi* hybrid form (PSU) established in the lab from eggs shipped from NIH from the G3 line in August 2015 (G3 strain hereafter).

All mosquitoes were maintained under standard conditions (12/12 h daylight/darkness light cycle, 27 ± 2 °C, RH 70% ± 10%). IRSS colonies were maintained by blood meals either on anesthetized rabbit hosts or at PSU by using bell jar feeders with either sausage casing or parafilm to hold a human blood meal mixed with CPDA-1 as an anticoagulant (sourced from either Valley Biomedical, Winchester, VA, USA or Biological Specialty Corp. Colmar, PA, USA). 10% (w/v) glucose sugar solution was provided for colony sugar feeding, with 0.05% of para aminobenzoic acid (PABA) at PSU and without PABA at IRSS.

#### Parasite isolates and strain

Natural parasite isolates and the NF54 strain were used in the experiments conducted at the IRSS and at PSU, respectively. At the IRSS, six parasite isolates collected from naturally gametocyte-infected patients in Burkina Faso were used to infect mosquitoes. These different isolates are named by capital letters A, B, C, D, E and F below. At PSU, mosquitoes were infected with NF54 gametocyte cultures originating from MRA-1000, MR4, ATCC Manassas Virginia.

#### Infectious feed

At IRSS, female mosquitoes were infected by using direct membrane feeding assays as previously described^36,37^. At PSU mosquitoes were infected using standard membrane feeding assays with NF54 *P. falciparum* parasite cultures following protocols described in^7^. Blood for culturing and infectious feeds was sourced from either Valley Biomedical (Winchester, VA) or Biological Specialty Company (Colmar, PA). Gametocyte cultures reached approximately 2–4% mature gametocytemia and were between 14 and 17 days post gametocyte induction when cultures were diluted with a mixture of freshly washed red blood cells and inactivated human serum and fed to 3–5 day old mosquitoes. Mosquitoes were maintained as in^7^ and after the 20 min infectious feed were immediately moved to incubators. Data loggers were used in each incubator and checked to confirm the accuracy of the incubators and adjust any offsets as required prior to the start of the experiments; monitoring continued for the duration of the experiments. Mosquitoes were provided with a sugar meal of 10% glucose on cotton wool following feeding.

### Mosquito dissections

Mosquitoes were dissected and midguts examined under 100× to 400× total magnification under phase contrast and/or dark field for microscopic examination of oocysts in dissected midguts (either stained with 1% mercurochrome at IRSS, or unstained at PSU) from 7 to 9 days post-blood meal (dpbm). Sporozoites were observed in dissected salivary glands at 1000× total magnification, also under phase contrast from days 12–14 at 27 °C unless other temperatures were specified.

### Salivary pathogens in transmission (“SPIT”) assay development

The assay used in the following experiments was optimized prior to use. Details about the assay development and optimization is provided in supplementary S1 data. This optimization process includes choosing the optimal substrate, minimum number of mosquito tested, and assay duration (S1, 1), DNA extraction method (S1, 2), and testing qPCR detection methods (S1, 3). Additionally, a standard curve was generated using final methods, and aspects of sample degradation were tested (S1, 4). All following experiments used the following optimized assay conditions: 15 mg cotton wools soaked in 10% glucose solution (either with or without PABA added) were left overnight on 28 mL plastic drosophila tubes hosting single mosquitoes. The cottons were collected the following morning, and DNA extracted using a slightly modified protocol provided in the Qiagen DNeasy Blood and Tissue kit. After DNA precipitation using absolute ethanol, the cotton wool substrate was transferred and only removed after DNA elution. Testing with known quantities of sporozoites under various conditions showed the malaria DNA can degrade with exposure to heat and humidity, so it is best practice to collect the sporozoites-containing cotton wool samples daily and store frozen until ready for DNA extraction (S1, 4). DNA amplification and visualization using qPCR SYBR methods that produced the best results are provided next; all other supporting information is provided in supplementary S1 data. Additionally, the utility of the assay at different temperatures was tested to determine if transmission to the sugar saturated cotton might occur at 12 °C, 14 °C, 16 °C, 18 °C, 32 °C, and 34 °C using sporozoite stage infected mosquitoes. At least some positive samples were detected at all temperatures except 12 °C, suggesting that if mosquitoes are active and sugar feeding, this assay could be functional; details provided in supplementary S2 data.

### qPCR detection of *Plasmodium falciparum*

Detection and quantification of the sporozoites in mosquito females and their saliva collected on cottons was performed by real-time PCR using standard procedures. We targeted the mitochondrial gene that codes for the cytochrome C Oxidase (Cox1)^38^. The sequences of the primers used were: qPCR-PfF 5′-TTACATCAGGAATGTTATTGC-3′ and qPCR-PfR 5′-ATATTGGATCTCCTGCAAAT-3′. Sample reaction occurred in a total volume of 10 μl containing 1 μl of DNA (~ 40 ng/μl); 4.6 μl of water; 2 μl of 1 × HOT Pol Eva Green qPCR Mix Plus ROX and 1.2 μl of each primer at 5 μM. The amplification began with an activation step at a temperature of 95 °C for 15 min and 40 cycles of denaturation at 95 °C to 15 s, annealing/extension at 58 °C for 30 s. Samples (cottons and female’s head/thorax) were considered positive for *P. falciparum* when the qPCR yielded a threshold cycle (Ct) < 35 and a 75 > Tm < 80 (at IRSS) or Ct < 35 and a 73 > Tm < 75 (at PSU). DNA extracted from salivary glands positives to *Plasmodium falciparum* (presence of sporozoites) was used as positive controls. Water samples and DNA extracted from cottons used to collect the saliva of uninfected mosquitoes (i.e. individuals that were not fed an infectious blood meal) were used as negative controls.

#### Comparing estimates of the parasite’s EIP using the classic dissection approach versus the non-destructive individual “SPIT” assay

At IRSS, *An. gambiae* mosquitoes were exposed to an infectious blood meal from two *P. falciparum* isolates (A = 168 gametocytes/µl of blood and B = 1 208 gametocytes/µl of blood) using direct membrane feeding assay. The extrinsic incubation period of *P. falciparum* was assessed through both microscopic observation of mosquito gut and salivary glands from 8 to 16 dpbm (i.e. the classic destructive approach) and the individual “spit” assay (non-destructive approach) over the same period of time.

##### Destructive approach: mosquito dissection and microscopic observation

From 8 to 16 dpbm, 8 to 20 mosquito females (median 14) exposed to isolate A or B were dissected daily. The presence and number of oocysts in mosquito guts and the sporozoites in the salivary glands were assessed microscopically. Oocyst rupture in mosquito midgut and sporozoite invasion of salivary glands is highly asynchronous. For instance at 12 dpbm, while some oocysts are intact and keep developing within a given mosquito gut, others have already ruptured and released their sporozoites (supplementary S3 data). To assess the timing of sporozoites dissemination in mosquito salivary, three metrics were derived from the microscopic observation:

(i) the proportion of infected mosquitoes with ruptured oocysts at 8–16 dpbm. This is the number of mosquitoes with at least one ruptured oocyst in their midguts out of the total number of infected mosquitoes (i.e. harboring either intact and/or ruptured oocysts);
(ii) the proportion of ruptured oocysts at 8–16 dpbm. This is, for each infected mosquito, the number of ruptured oocysts out of the total number of oocysts (intact + ruptured);
(iii) the proportion of oocyst-infected mosquitoes with sporozoites in their salivary glands at 8–16 dpbm. This is the number of oocyst-infected mosquitoes harboring sporozoites in their salivary glands out of the total number of infected mosquitoes (i.e. harboring either intact and/or ruptured oocysts). Following microscopic observation, salivary glands were stored at − 20 °C in individual 1.5 mL Eppendorf tube for further qPCR determination of infection status; such that we were able to compare microscopic and molecular diagnostic for this metric.

##### Non-destructive approach: the individual “SPIT” assay

Twenty one (21) females per parasite isolate (A or B) were individually placed in 28 mL plastic drosophila tubes at 7 dpbm and cotton balls (15 mg/piece) soaked with 10% glucose solution were placed on the tube gauze at 17:00 until collection at 7:00 in sterile 1.5 mL Eppendorf tubes stored at − 20 °C for further qPCR analysis. Cottons were replaced daily from 7 to 15 dpbm and saliva was collected from 8 to 16 dpbm. At the end of the experiment, the presence of parasites in the heads/thoraxes of the females used to collect mosquito saliva was tested using qPCR as described above. Individual EIP was defined as the time between the infectious blood meal and the first day of positive molecular detection by qPCR of *P. falciparum* from a cotton wool substrate collected for a given female.

#### Individual estimation of EIP in different mosquito species

##### IRSS

Mosquito females of *An. gambiae*, *An. coluzzii*, and *An. arabiensis* were all exposed to an infectious blood meal from one volunteer carrying gametocytes (isolate C: 3024 gametocytes/µl of blood) using direct membrane feeding assays. At 7 dpbm, 20 females from each species were individually placed in plastic drosophila tubes for saliva collection using the spit assay as described above. In this experiment, saliva was collected daily from 8 to 14 dpbm and every other day from 14 to 20 dpbm and stored at − 20 °C before DNA was processed.

##### PSU

From 6 dpbm cotton wool substrate was provided the same way as in experiment 2.1. to 15 individuals of *An. stephensi* that were fed on blood infected with the NF54 *P. falciparum* parasite culture. Cotton balls were left on cups from 14:30 until collection at 09:30 and replaced daily from 7 to 21 dpbm. Cotton balls were collected and stored for up to 1 week at − 20 °C before transferring to − 80 °C until DNA isolation and qPCR.

#### Determination of mosquito sugar feeding rate

##### IRSS

Assay 1*:* Four-day-old females of *An. gambiae*, *An. coluzzi* and *An. Arabiensis* received an uninfectious meal from rabbit blood. Two days later, 30 females from each species were individually placed in 180 mL plastic cups with white filter paper at the bottom. Every evening at 17:00 cotton balls soaked with 10% glucose and containing a blue dye (1 mg mL^− 1^ Fast Green FCF/Xylene cyanole FF; Sigma-Aldrich)^39^ were deposited. The day after at 07:45, the cottons were removed from the plastic cups. At 16:00, the presence or absence of blue droppings on the paper was observed. The presence of dejection on the paper indicates that a sugar intake by the mosquito occurred and the absence of dejection is considered us no sugar intake. The same day at 17:00, new cottons were added to the same plastic cups, but with a 10% (w/v) glucose solution containing a yellow dye (1 mg mL^−1^ Acid Yellow 17; Sigma-Aldrich, St Louis, Missouri). This procedure was repeated during 8 consecutive days with the papers changed every day and blue and yellow dyes on cottons switched between days.

Assay 2: To determine whether *P. falciparum* infection influenced the frequency of mosquito sugar feeding, *An. coluzzi* females were exposed to infectious blood from a naturally-infected volunteer (parasite isolate D: 696 gametocytes/µl of blood). On 7 dpbm, the level of oocyst infection was measured by dissecting the midguts of 30 females. The proportion of infected females was 0.9 (27/30) with a mean number of 33 ± 5 oocysts. On the 13th day post infection, 30 females were individually placed in plastic cups with white filter paper at the bottom. The same day at 17:00 the cotton balls soaked with 10% glucose solution containing a blue dye (1 mg mL^−1^ Fast Green FCF/Xylene cyanole FF; Sigma-Aldrich) were placed on top of the cup gauze. The next day at 07:00, the colored cottons were removed and at 16:00 the presence or absence of mosquito droppings on the white filter paper were recorded. The same day at 17:00 this procedure was repeated with the same mosquitoes, but with a 10% glucose solution containing a yellow dye (1 mg mL^−1^ Acid Yellow 17; Sigma-Aldrich, St Louis, Missouri)^39^. The papers were changed every day and blue and yellow dyes were switched between days. The color of the fecal dots (blue, yellow or green, when two consecutive sugar meals were mixed up) were also specified each day at 16:00. The assay lasted 11 days until 24 dpbm. Females that died before 17 dpbm (i.e. from which less than 3 colorimetric measures could be performed were excluded). At the end of this experiment, we obtained 16 females surviving beyond 17 dpbm and from which were gathered 3 to 11 (median = 5) individual colorimetric measures. DNA present on cotton samples and carcasses of these 16 females was isolated and sporozoites quantified as in experiments 1 and 2.

##### PSU

Fifteen *An. stephensi* were individually housed and fed with colored sugar solution by providing 2 mL of sugar and dye added to medium size cotton wool pads (750 µl of blue food dye mixed with 15 mL 10% glucose solution). The cotton wool was held in place in the cap of plastic tube, glued to the bottom of a clear plastic cup, which was inverted over filter paper. The filter paper was held in place by a petri dish lid underneath to prevent mosquito escape from this cage and changed daily at 15:00 each day for 1 week. Mosquitoes were knocked down for 2.5 min in the freezer (− 20 °C) to sort them randomly into cups. The 15 mosquitoes used in the experiment were previously fed non-infectious human blood at 6 days of age. These mosquitoes were used three days after their blood meal to encourage complete digestion and monitored daily thereafter. Color dots from fecal material from 15 *An. stephensi* individuals were counted on the daily filter paper samples.

A second experiment was conducted to test the effect of temperature on sugar feeding in two species as estimated by fecal dot production (a measure of digestion rate). A set of 45 *An. stephensi* and 45 mosquitoes of the G3 strain were treated the same way and set up at 9 days of age in inverted plastic cups as before. These were further divided into three temperature treatments with 15 mosquitoes per treatment and were housed at either 20 °C, 27 °C, and 32 °C and monitored for colored fecal dot production for eight days. Blue or green artificial food dye was used because pilot tests using 5 colors of various natural and artificial food dyes (as well as no dye) for sets of 5 *An. stephensi* mosquitoes housed and maintained in this way showed while there was no effect of any dye (compared to no dye) on mortality, the green and blue artificial food dyes were easier to see in fecal dots. Red dye resulted in fewer fecal dots (either from lack of feeding or a difference in processing) and yellow and natural red derived from beets were slightly more difficult to see.

#### Infection duration

Both at IRSS and at PSU, mosquitoes were tested for their ability to expel sporozoites on sugar cottons throughout their lives.

##### IRSS

*Anopheles gambiae* and *An. coluzzii* mosquitoes received an infectious blood meal from one of two *P. falciparum* isolates (E: 176 gam/µl blood and F: 112 gam/µl). Seven dpbm, mosquitoes were placed in individual tubes for mosquito saliva collection and followed individually until all individuals died. Thirty-four cotton samples were collected between 23 and 54 dpbm from 4 *An. coluzzii* individuals (1 infected with isolate E and 3 infected with F) and 3 *An. gambiae* individuals (1 infected with isolate E and 2 infected with F). These “late infection” cottons (23–54 dpbm) were used for DNA extraction using the Qiagen technique followed by qPCR amplification as described above.

##### PSU

*Anopheles stephensi* mosquitoes, infected with the NF54 *P. falciparum* parasite culture as previously described, were kept in cups of either: a single mosquito, group of 4, group of 5, or a large group of more than 100, respectively. Mosquitoes had sugar soaked cotton substrates placed on top of the cups from 14:00 to 9:30. Cottons were collected and changed daily from 1 to 40 dpbm, and analyzed intermittently out to 40 dpbm. At 40 dpbm all surviving mosquitoes were dissected and the terminal cottons from 40 dpbm were subjected to molecular analysis.

An additional 43 infectious *An. stephensi*, maintained individually, were sampled for the presence of parasites in the saliva every 5 days from 35 dpbm until death using sugar soaked 15 mg cotton pads. Mortality was such that final sampling included 30 dpbm, N = 43; 35 dpbm, N = 36; 40 dpbm, N = 21; 45 dpbm, N = 11; and 50 dpbm, N = 3, at which point the experiment was terminated. No accompanying dissections were performed on these individuals, but from other data collected from mosquitoes fed under the same conditions, infection prevalence was nearly 100% and intensity of sporozoites was high at 15 days post infection. Extraction and detection of parasite DNA from both assays of the 4B experiment was done from the 15 mg cotton samples as described above.

### Statistical analyses

All statistical analyses were performed using R (version 4.0.2)^40^. Logistic regression by generalized linear models (GLM, binomial errors, logit link, or quasibinomial errors) were used to test the effect of parasite isolates and day post blood meal (dpbm) on (i) the proportion of infected mosquitoes with ruptured oocysts, (ii) the proportion of ruptured oocysts, (iii) the proportion of oocyst-infected mosquitoes with sporozoites in their head and thorax. Since the outcome variable as part of the “spit” assay is the time when sporozoites are first detected in cottons from individual mosquito, we used the statistical approach specifically developed for investigating the time a specified event takes to happen. Cox proportional hazard models were therefore performed to test for statistical difference in sporozoites appearance time (or EIP by extension) among parasite isolates and mosquito species. We also analyzed the relationship between the EIP and the lifespan of infected mosquitoes using a generalized linear model (GLM). Model simplification used stepwise removal of terms, followed by likelihood ratio tests (LRT). Term removals that significantly reduced explanatory power (P < 0.05) were retained in the minimal adequate model^41^.

### Ethics declarations

#### Approval for human experiments

The protocol for the recruitment of patients (children aged 5 to 12 years) naturally infected by gametocytes, was approved by the IRSS (Institut de Recherche en Sciences de la Santé) ethics committees (2017-003/MESRSI/CNRST/IRSS/CEIRES). Prior to inclusion, the informed consent of the volunteers' parents or legal guardians was obtained. All experiments were conducted in accordance with applicable guidelines and regulations.

#### Approval for animal experiments

Animal work was conducted under license and approval from the Office of Laboratory Animal Welfare of US Public Health Service (Assurance Number: A5928-01) and the National Committee of Burkina Faso (IRB 00004738 and FWA 00007038). All experiments with rabbits were carried out in strict accordance with the recommendations in the Guide for the Care and Use of Laboratory Animals of the National Institutes of Health. Additionally, the study was carried out in compliance with the ARRIVE guidelines. Trained personnel and veterinarians cared for all animals involved in this study.

## Results

### Comparing estimates of parasite’s EIP between the classic dissection approach and the non-destructive individual “spit” assay

#### Destructive approach: mosquito dissection and microscopic observation

A total of 121 mosquito females exposed to parasite isolate A and 114 to isolate B were dissected from 8 to 16 dpbm (between 8 and 20 females/day, median = 14) to assess microscopically the presence and number of oocysts in the midguts and of sporozoites in salivary glands. Salivary gland infections were also confirmed through qPCR. The infection rate was high with 117/121 (96.7%) and 114/114 (100%) of females exposed respectively to isolate A and B harboring parasite oocysts in their midguts (supplementary S44, Fig. S4a). The gametocytemia of isolate B (1208 gam/µl) was higher than that of isolate A (168 gam/µl), resulting in strong difference in the number of developing oocysts between the two isolates (B: 191.65 ± 21, A: 13.86 ± 2, supplementary S4, Fig. S4b, *LRT X*^*2*^_*1*_ = 24.46, P < 0.001). Similar patterns of infection were observed in salivary glands for the sporozoite stages (supplementary S4, Fig. S4c,d).

**Figure 1:**
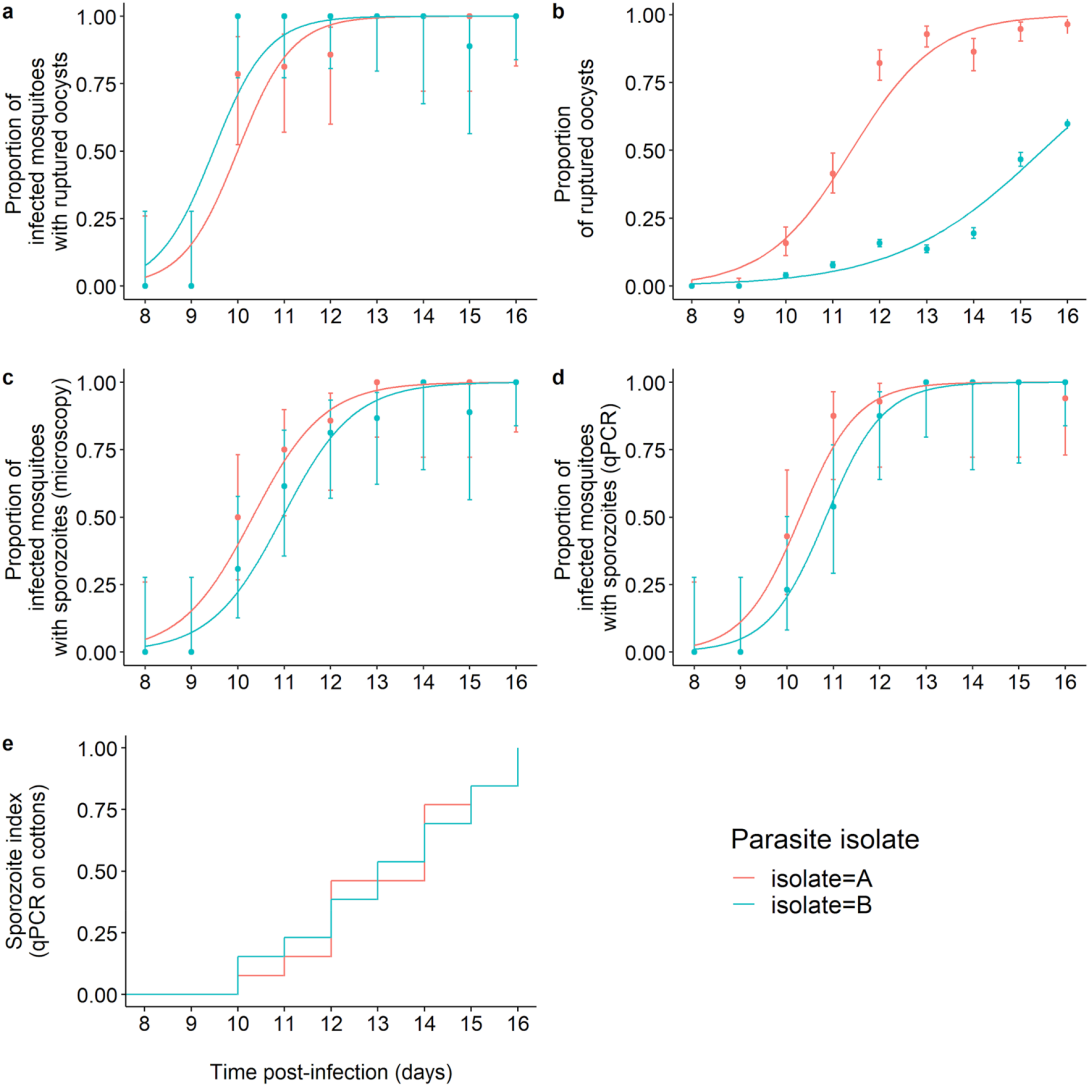
The extrinsic incubation period of *Plasmodium falciparum* estimated using classical dissection approaches (**a**–**d**) and a novel non-destructive assay (**e**). (**a**) Proportion of infected mosquitoes with ruptured oocysts (± 95% CI) from 8 to 16 dpbm, expressed as the number of mosquitoes with at least one ruptured oocyst out of the total number of infected mosquitoes (i.e. harboring either intact and/or ruptured oocysts) for two parasite isolates. The lines represent best-fit logistic growth curves for each isolate. (**b**) Proportion of ruptured oocysts (± 95% CI), expressed as the number of ruptured oocysts out of the total number of oocysts (intact + ruptured). The lines represent best-fit logistic growth curves for each isolate. (**c**) Proportion of oocyst-infected mosquitoes with microscope-identified sporozoites in the salivary glands (± 95% CI), expressed as the number of oocyst-infected mosquitoes harboring sporozoites in their salivary glands out of the total number of infected mosquitoes. The lines represent best-fit logistic growth curves for each isolate. (**d**) Same as (**c**) but the presence of sporozoites was detected using qPCR. (**a**–**d**) Sample size = 8 to 20 midguts /day/isolate (median = 14). (**e**) Kaplan–Meier curves representing the temporal dynamics of sporozoites appearance in small pieces of cotton used to collect saliva from individual mosquitoes.

The few uninfected mosquitoes (N = 4 individuals from isolate A) were excluded from the analysis of the EIP. None of the dissected mosquitoes at 8 and 9 dpbm exhibited ruptured oocysts and the first observations occurred at 10 dpbm for both isolates. As expected, there was a highly significant positive relationship between time post-bloodmeal and the proportion of mosquitoes with ruptured oocysts (*LRT X*^*2*^_*1*_ = 147, P < 0.001, Fig. 1a). The timing of rupturing was similar between the two parasite isolates (*LRT X*^*2*^_*1*_ = 1.39, P = 0.24, Fig. 1a). Using this metric, the estimated EIP_50_ from the binomial model was 9.98 days for isolate A and 9.49 for isolate B.

The proportion of ruptured oocysts was higher in mosquitoes exposed to isolate A than B (A: 879 ruptured oocysts out of 1503 counted oocysts (58.5%) from 8 to 16 dpbm, B: 3589/19,035 (18.9%) over the same period of time, *LRT X*^*2*^_*1*_ = 1045, P < 0.001, Fig. 1b). This suggests a negative effect of density on parasite growth at the oocyst level, while no such density-dependent effect was observed on the proportion of mosquitoes with ruptured oocysts (Fig. 1a) or with disseminated sporozoites (Fig. 1c,d). To confirm this hypothesis, the relationship between the proportion of ruptured oocysts and the total number of oocysts was explored at the mosquito individual level (n = 190 mosquitoes following the exclusion of the 8 and 9 dpbm time points for which there was no ruptured oocysts). There was a strong negative relationship (*LRT X*^*2*^_*1*_ = 16, P < 0.0001, Fig. S4.2): the smaller the number of developing oocysts in mosquito guts, the greater the fraction of ruptured oocysts from 10 to 16 dpbm was.

Finally, the proportion of mosquitoes with disseminated sporozoites in their salivary glands, the most epidemiologically-relevant metric, did not vary between parasite isolate, be it measured through microscopic observation (*LRT X*^*2*^_*1*_ = 0.96, P = 0.33, Fig. 1c) or qPCR (*LRT X*^*2*^_*1*_ = 0.71, P = 0.40, Fig. 1d). The proportion of sporozoite-infested salivary glands increased with dpbm similarly for both parasite isolate (i.e. no significant dpbm by isolate interaction) and regardless of the method used. Using this metric, the estimated EIP_50_ from the binomial models were 10.35 (microscopy) and 10.26 days (qPCR) for isolate A and 10.93 (microscopy) and 10.85 days for isolate B.

The estimated time required for the sporozoites to migrate and invade the mosquito salivary glands following the egress from the oocysts was thus 9 h for isolate A (i.e. EIP_50_ derived from the sporozoites observation in salivary glands (Fig. 1c)—EIP_50_ derived from the oocyst rupturing data (Fig. 1a): 10.35 days–9.98 days), and 34 h (10.93 days–9.49 days) for isolate B.

#### Non-destructive approach: the individual “spit” assay

Forty-two (42) females (21 per parasite isolate) were individually placed in tubes for saliva collection from 8 to 16 dpbm. Mosquito survival over the collection period is described in the supplementary S4 (Fig. S4e). At the end of the experiment on 16 dpbm, 37 females (17 in A and 20 in B) were identified as infected using qPCR. Of these, 26 (13 in each isolate) produced cottons containing detectable traces of parasite DNA by qPCR (i.e. positive cottons). The infected females that did not produce any positive cottons from 8 to 16 dpbm (4 females for isolate A and 7 for B) were excluded from the analysis because no EIP values can be derived from these samples. A total of 214 cottons (112 for A and 102 for B) collected from 26 females were thus analyzed.

Similar to microscopic observation, the first positive cottons occurred on day 10 for each isolate. The parasite EIP_50_ at 27 °C using this assay was 14 days for isolate A and 13 days for isolate B (isolate effect: *LRT X*^*2*^_*1*_ = 0.001, P = 0.97, Fig. 1e).

### Individual estimation of EIP in different mosquito species

#### IRSS

The saliva of 20 *An. arabiensis*, 20 *An. coluzzii*, and 20 *An. gambiae* fed with the blood from a naturally infected gametocyte carrier (parasite isolate C) was collected using the “spit” assay from 8 to 20 dpbm. Of these, 18 *An. arabiensis*, 17 *An. coluzzii, and* 20 *An. gambiae* were confirmed as infected using qPCR on carcasses of dead mosquitoes. Cottons collected from uninfected females (N = 5) were discarded. Two infected females (one *An. gambiae* and one *An. coluzzii*) died at 7 dpbm before the collection of saliva has started (full survival results are given in supplementary S5, Fig. S5.2). A total of 368 cotton samples from 53 females (18 *An. arabiensis*, and 16 *An. coluzzii*, 19 *An. gambiae*) were therefore analyzed using qPCR. The proportion of positive cotton balls and the proportion of mosquitoes generating positive cotton samples are given in Table 1 for each species. Over the collection period from 8 to 20 dpbm, 19 individuals (2 *An. arabiensis*, 10 *An. coluzzii* and 7 *An. gambiae*), of the 53 infected females used to collect the saliva, never generated positive cotton samples (Table 1). This was mainly due to early mortality prior to the sporozoites invasion of mosquito salivary glands as this number fell to three (one from each mosquito species) between 13 and 20 dpbm.

**Table 1:** Proportion of mosquitoes producing at least one positive cotton over the collection period and proportion of positive cottons both over the collection period and after the first positive detection (“post-EIP”) for each anopheline species.

Proportion of:	Collection period	*Anopheles arabiensis*	*Anopheles coluzzii*	*Anopheles gambiae*	*Anopheles stephensi*
Infected mosquitoes generating at least one positive cotton	8–20 dpbm	0.89 (16/18)	0.38 (6/16)	0.65 (12/19)	0.93 (14/15)
Positive cottons	8–20 dpbm	0.26 (40/152)	0.08 (7/93)	0.33 (40/123)	0.52 (99/190)
Positive cottons	Post EIP	0.69 (40/68)	0.27 (7/26)	0.71 (40/56)	0.81 (99/122)

**Figure 2:**
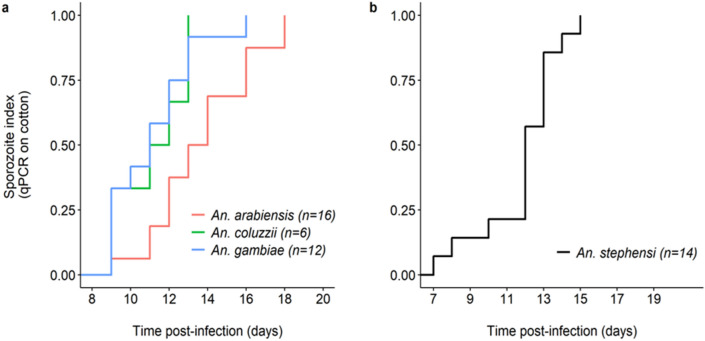
The extrinsic incubation period of *Plasmodium falciparum* in four *Anopheles* mosquito species. (**a**) Kaplan–Meier curves representing the temporal dynamics of sporozoite appearance in small pieces of cotton used to collect saliva from individual mosquitoes in the three major African vectors *An. arabiensis* (red)*, An. gambiae* (blue) *and An. coluzzii* (green). (**b**) Same as (**a**) but for *An. stephensi*. The numbers in brackets indicate the number of females for each species of mosquito that generated at least one positive cotton. African vectors were infected with the *P. falciparum* isolate C and *An. stephensi* with the NF54 laboratory culture.

EIP, defined as the time between the infectious blood meal and the first day of positive molecular detection by qPCR of *P. falciparum* from the cotton used to collect saliva, varied among species (LRT *X*^*2*^_2_ = 8, P = 0.018, Fig. 2a). The shortest EIP_50_ was observed in *An. gambiae* (11 days, min: 9, max: 16), followed by *An. coluzzii* (11.5 days, min: 9, max: 13) and *An. arabiensis* (13.5 days, min: 9, max: 18). Following parasite invasion of their salivary glands (i.e. the day when parasite DNA were first detected in cottons), individual females did not systematically generated positive cotton samples (Table 1). The “proportion of positive cottons post-EIP” refers to the sensitivity of our assay; that is, its ability to correctly detect parasite DNA in cotton samples collected on the days following the first day of positive detection. In other words, this is the proportion of cotton samples that tested positive for *P. falciparum* among those that were used to collect saliva of females that previously generated a positive cotton. Sensitivity was 0.69, 0.27, and 0.71 for *An. arabiensis, coluzzii,* and *gambiae* respectively (Table 1). Thus, *An. coluzzii* tended to deposit detectable quantity of sporozoites on cottons less frequently than the two other mosquito species following the parasite’s EIP.

A major driver of cotton positivity rate following parasite’s invasion of salivary gland was the infection intensity in females used to collect the saliva. First, *An. coluzzii* mosquitoes, the species with the lowest sensitivity (Table 1), also displayed lower infection intensity than *An. arabiensis* and *An. gambiae* (supplementary S5, Fig. S5.1a). Second, there was a positive relationship between the probability to generate Pf-positive cotton samples and infection intensity in individual females (LRT X^2^_1_ = 10, P = 0.002, Fig. S5.1b), regardless of mosquito species (species by sporozoite intensity (Ct) interaction: LRT X^2^_1_ = 1.9, P = 0.4, Fig. S5.1b). However, there was no relationship between the Ct of females used to collect saliva and the Ct of positive cottons (LRT X^2^_1_ = 1.9, P = 0.4).

#### PSU

Parasite-positive cotton samples were detected in *An. stephensi* (PSU). Of the 15 mosquitoes sampled, 14/15 *An. stephensi* individuals generated positive cotton samples at least once during the sampling period. After the dissection of all surviving mosquitoes at the end of the experiments, it was found that 12/12 *An. stephensi* were infected with salivary glands found harboring sporozoites. EIP50 similarly defined as in 2a was 12 days (Fig. 2b).

**Figure 3:**
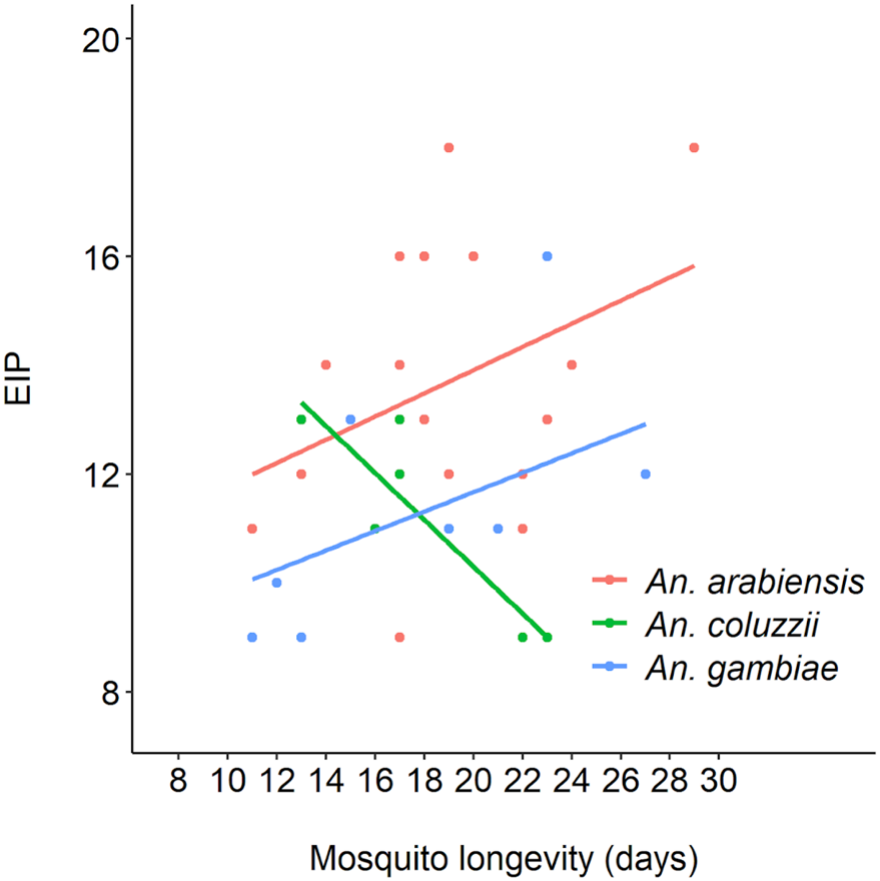
Relationship between the extrinsic incubation period (EIP) of *Plasmodium falciparum* and the lifespan of individual mosquitoes from three mosquito vector species.

The spit assay makes it possible to study the links between different traits at the mosquito individual level. For the 2A IRSS experiment, cotton samples were collected daily up to 20 dpbm but the females used were kept in tubes until their death, thus making it possible to link the EIP with mosquito lifespan at the individual level. There was a significant positive correlation between EIP and mosquito longevity (LRT X^2^_1_ = 21, P = 0.035, Fig. 3) such that short EIPs were also associated with short mosquito lifespan. There was no mosquito longevity by species interaction on EIP (LRT X^2^_2_ = 25, P = 0.07, Fig. 3), although 6 observations in *An. coluzzii* (very low statistical power) pointed to a negative correlation in this species.

### Determination of mosquito sugar feeding rate

#### IRSS

##### Assay 1

The overall mosquito survival rate during the 8 days-assay was 89%, with *An. gambiae* dying at a faster rate than both *An. arabiensis* and *An. coluzzii* (8/30 vs 0/30 and 3/30, *X*^*2*^_2_ = 11, P = 0.004). Dead mosquitoes were excluded from the analysis. A meal was scored as having been taken when filter papers at the bottom of the cups contained either blue, yellow or green fecal dots. The papers were changed daily and the presence of colored fecal dots was scored daily. Of the 79 mosquito survivors, 31 individuals generated colored filter papers all 8 collection days. The average number of positive days was 6.89 ± 0.14 (min: 2 days, median: 7 days, max: 8 days). The overall detected sugar feeding frequency over this period was 544 meals/632 feeding opportunities = 86%. The sugar feeding rate of *An. arabiensis* and *An. coluzzii* females was higher than that of *An. gambiae* (89%, 91% and 76% respectively, GLMM with mosquito identity considered as a random effect: *X*^*2*^_2_ = 13.9, P < 0.001).

##### Assay 2

The 16 females used to assess the presence of colored fecal dots from 14 to 24 dpbm were all infected (mean Ct = 22.2 ± 1.37). A meal was scored as positive when filter papers at the bottom of the cups contained either blue, yellow or green fecal dots. A total of 91 colored cottons soaked in 10% glucose were retrieved from these infected females over the collection period, of which 63 were positive to *P. falciparum* (i.e. a 69% sensitivity) (Table 2, supplementary S6). The color of the cotton (yellow or blue) had no incidence on the probability to detect *P. falciparum* (*X*^*2*^_1_ = 0.008, P = 0.93). Colored dots were observed on 61 filter papers of a total of 91 observations; that is, a daily feeding frequency of 67%.

**Table 2:** Association between sugar feeding (presence of colored dots) and production of Pf-positive cotton samples (cotton Pf-positivity).

	Presence of colored dots		P-value
	Yes	No	
**Cotton Pf-positivity**			
Yes	40	23	0.40
No	21	7	

Cotton Pf-positivity: yes (positive cotton) and no (negative cotton). Presence of colored dots: yes (presence of colored dots) and no (absence of colored dots). Pf indicate *Plasmodium falciparum*. P = 0.40, no association between sugar feeding and cotton Pf-positivity.

Mosquitoes expectorating sporozoites of *P. falciparum* on cotton pads during the night (as evidenced by Pf-positive cotton samples, n = 63) egested colored droppings on 40 occasions (63.4%). Contrary to our prediction, the absence of *P. falciparum* in cottons was not necessarily associated to the absence of colored dots: there were 21 occurrences of parasite-negative cotton samples but positive sugar meals, and only 7 occurrences of parasite-negative cotton samples and negative sugar meals (*X*^*2*^_1_ = 0.69, P = 0.40, Table 2). Starting from dpbm 15, there was a high proportion of green dots or combinations of green + yellow dots or green + blue dots. This shows that colored dots observed at 16:00 on a given day did not necessarily come from the digestion of a sugar meal taken during the same night, but could also result from previous sugar meals, meaning that mosquito sugar digestion can last > 24 h. These results suggest that measuring colored fecal dots does not reliably provide evidence of either sugar feeding by night, or of transmission of parasites to cotton pads.

#### PSU

The 15 *An. stephensi* mosquitoes maintained at 27 °C for 1 week produced sugar fecal dots most of days, suggesting a regular feeding. Due to some mortality, there were only 79 sample-days, but on 66 of these days mosquitoes produced at least 4 or more new dots on filter paper, suggesting they were likely feeding that day on the dyed sugar meal about 83.5% of days. No dots were observed on 8 sample-days, or 10.1% of the time, with some variation of days with fewer than 4 dots observed making up the difference, where it was unclear if they were producing new dots or still digesting the previous day’s meal.

Color fecal dot production was monitored for *An. stephensi* (8 days) or G3 strain mosquitoes (7 days) housed at either 20 °C, 27 °C, and 32 °C. As might be expected, G3 strain mosquitoes produced fewer fecal dots at lower temperatures and greater numbers of dots at high temperatures. *An. stephensi* showed a similar pattern, although mortality at 32 °C was very high in this test which could have affected average dot production due to some individuals dying before digestion was completed (Table 3).

**Table 3:**
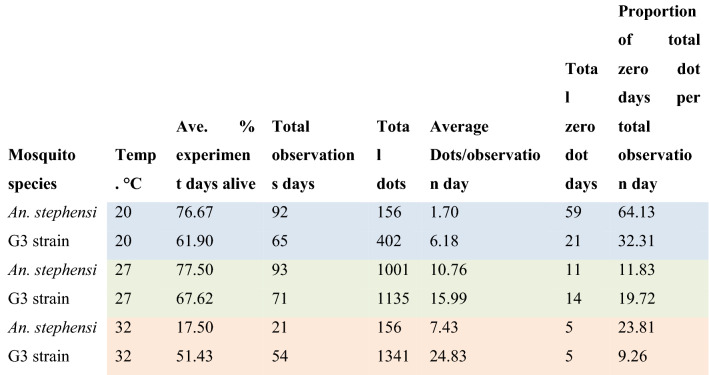
Summary of sugar feeding results comparing two species at three temperatures.

The shaded values provided in Table 3 are important because each color represents a given temperature. The blue color refers to the results of the presence of dotes for mosquitoes (both species) kept at 20°C. The green color is the results for mosquitoes kept at 27°C and the orange color is for mosquitoes kept at 32°C.

Generally, these results suggest that using the sugar-feeding assay to detect parasites will be more effective at warmer temperatures, and more false negatives are predicted at lower temperatures if mosquitoes are feeding less often. Around typical temperatures used in many infection studies (27 °C) mosquitoes are estimated to have fed about 80–90% of days sampled.

### Infection duration

#### IRSS

From a total of 34 cotton balls collected from 25 to 53 dpbm, 17 were positive. We could detect *P. falciparum* sporozoites in old cotton samples from 23, 26, 33, 36 and 39 dpbm.

#### PSU

For samples at PSU tested in groups of 1, 4, 5, or 100+ in cups, surviving *An. stephensi* were carried through until day 40 dpbm, although only a few of these samples were analyzed. From this, there was evidence of salivary gland infection and transmission to cotton substrate as late as day 40 post infection from a group of 3 (those remaining from a set of 5), and also the larger group of 100, which by this point had about 20 mosquitoes remaining. Dissected mosquitoes were all found to have sporozoites in their salivary glands at 41 days post infection. This is evidence that infection can persist at least until day 40 post infection, and possibly until mosquito death.

## Discussion

Collectively, these data support that the assay we have developed is capable of monitoring individual mosquitoes repeatedly for infection status at transmission stages of malaria. We have further tested its robustness for use across various temperatures, finding it is functional ranging from 14 to 34 °C, and show that mosquitoes can remain infectious and continue to transmit parasites well into their old age, out to 40 days post-infection. The assay described here compared favorably to other assays with similar goals of passive monitoring^27^. In another study, at 21 and 24 dpbm, respectively 31% and 55% of cotton wools collected from tubes hosting three *P*. *berghei-*infected females were positive^27^. With mosquitoes kept individually, we found a detection rate (sensitivity) ranging from 0.27 to 0.81 depending on mosquito species (Table 1). The sensitivity of the presented assay was influenced by the amount of sporozoites in the salivary glands (supplementary S5 data). This may explain the observed inter-specific variation in sensitivity, with *An. coluzzii* displaying the lowest infection intensity and sensitivity (supplementary S5, Fig. S5.1a). To reach high level of sensitivity, we therefore recommend monitoring highly-infected females with this technique. The assay is easy and simple, only requiring basic molecular techniques of DNA extraction using a standard kit, and simple qPCR, a highly sensitive method for detecting the Cox1 gene in as few as six sporozoites^42^. There has been much demand to determine whether a mosquito is transmitting sporozoites without dissecting it; including within human malaria research in areas as diverse as monitoring transmission in the field to developing better vaccines^15,25–27,43^. It can also be useful for animal models of malaria in the lab^44^. Additionally, it has the potential to advance studies on wild mosquito populations in the field on less-well understood species of avian malaria for example, where estimating infection prevalence in wild-caught mosquitoes when the timing of infection is typically unknown, or for generating experimental infections to examine host specificity in the lab if it is unclear if a mosquito is infectious since timing of EIP can vary by malaria species^45^. Applications of this low-tech innovation are many, and the most useful will be determined by those working in *Plasmodium* research and looking to avoid long hours of dissecting mosquito after mosquito to determine transmission, or through necessity if infected mosquitoes are limited. It would also open up the field to less-skilled/dexterous workers to aid in research as adding and removing sugar soaked cotton is non-technical, and extractions and qPCR routine compared to developing expertise at dissecting and scoring infections through microscopy.

Limitations of this non-destructive assay include a possible overestimation of the parasite’s EIP_50_ compared to classical measures using microscopic observations of salivary glands. In Experiment 1, the estimated EIP_50_ at 27 °C of *P. falciparum* was 10 to 11 days using microscopy and 13 to 14 days with the non-destructive approach. Estimates derived from the second experiment using the non-destructive technique were shorter with EIP_50_ of 11 to 13 days depending on the vector species. The longer EIP estimates obtained with individual monitoring using the spit assay could be explained by the existence of false negative cotton samples. First, because of sensitivity < 100%, infectious females may deposit undetectable level of sporozoites onto cottons. This is especially true at the onset of salivary gland invasion when the amount of sporozoites is relatively low. Another source of false negatives is the biology of mosquito sugar feeding. Mosquitoes do not feed daily on sugar 100% of the time, and on average about 20% of potentially positive samples will be missed just because of skipped sugar feeds by infectious females within a 24 h period, resulting in false negatives. This risk can be partially mitigated by feeding dyed sugar water and determining if mosquitoes are feeding at all by looking for colored fecal dots on the cage floor. If there are no dots for a few days, it is unlikely the mosquito is feeding on the cotton or that the sample will be positive for sporozoites (though digestion time will vary with temperature so the correlation is not perfect). Finally, sporozoites typically clump together and can be transmitted in groups in saliva^46^. This is further evidenced in observations by Frischknecht and collaborators^47^ where sporozoites were observed to move in clumps into salivary ducts for transmission. This suggests that transmission can be spotty and unpredictable by nature, even in infectious mosquitoes, yet this may be adaptive for the parasite if it allows the sporozoites to remain below a detection threshold in the vertebrate host upon inoculation, yet have enough transmitted at a time to establish infection^43,46,48^. It is also worth mentioning that to avoid wasting time on a feed that was not infectious, it is always best to check at least a few mosquitoes for oocysts and/or sporozoites using dissection at appropriate time points to ensure the infection was successful.

Longer EIPs values obtained using the spit assay compared to microscopic observations (experiment1) could also result from the existence of a delay between the sporozoite invasion of salivary gland (the trait measured by microscopic observations) and the expulsion of sporozoites in saliva (the trait measured by the spit assay). For example, in *Aedes aegypti* infected with *Plasmodium gallinaceum*, only about 80% of infectious mosquitoes released sporozoites during a forced salivation assay, and it was observed that there was a one-day delay between the appearance of parasites in the salivary glands and the sporozoites being released with saliva^49^. The difference between EIP values derived from microscopic observations and that from the detection in cotton samples therefore supports the existence of such a delay in *P. falciparum*-infected *Anopheles* mosquitoes. This experiment also showed that the first ruptured oocysts occurred at 9–10 dpbm and that the average time between oocyst rupture and the first invasions of salivary glands was 9 to 34 h depending on the parasite isolate. The rate of oocyst rupturing was recently considered as the major driver of EIP and hence a key epidemiological parameter^50^. Our observations suggest that the day of first oocyst rupture + 1 may then roughly indicate the time of salivary gland invasion. Moreover, we confirm that only a fraction of oocysts ruptures to release sporozoites and that this fraction seems to depend strongly on the oocyst intensity in the gut (Fig. 1b, Fig. S4.2). Our results support recent findings showing that oocysts can enter a dormancy-like state under crowding conditions^51^. Further work is needed to better understand variations in the delays between both the oocyst rupturing and salivary gland invasion and between invasion and injection with saliva.

Mosquito survivorship with the assay was high and long-term individual tracking revealed that sporozoite transfer onto cotton wools can persist at least until day 40 post-infection. Given the relatively short lifespan of *Anopheles* mosquitoes in field conditions (15–20 days)^52^ this result therefore suggests that mosquitoes likely remain infectious for life.

Perhaps the greatest utility of the assay is that it can be used for individual mosquitoes. Currently, nearly all estimates of sporozoite prevalence, or even estimates of EIP, are measured as averages of groups of mosquitoes being sampled. Even taking into account the possibility for false negatives using the assay, there are great opportunities to monitor aspects of effects of malaria infection on mosquitoes that we have not previously had the chance to explore. This could open up areas of research to look at parasite transmission over lifespan, individual variability in earliest transmission, possibilities to examine how fecundity is (or is not) affected by infection, and associations between transmission traits such as e.g. EIP, infection level, survival and fecundity. As one example, we found a positive relationship between mosquito lifespan and the parasite’s EIP, suggesting the existence of a trade-off between two important transmission traits. A similar pattern was previously observed with a non-destructive sugar-feeding assay in the dengue—*Aedes aegypti* association^29^. The trade-off between EIP and mosquito longevity could be mediated through parasite factors (e.g. more virulent parasite isolates also develop faster) and/or mosquito factors (e.g. poorly vigorous mosquitoes with low lifespan perspective are also more permissive to the development of the parasite). Additional work will be required to confirm this relationship. The non-destructive sugar-feeding assay developed here is a unique opportunity to quantify concomitantly multiple parasite and mosquito traits at the individual level and hence will contribute to a better understanding of the evolution of key epidemiological traits.

## Supplementary Information

Supplementary Information.
