# Supplementary material for: A non-destructive sugar-feeding assay for parasite detection and estimating the extrinsic incubation period of Plasmodium falciparum in individual mosquito vectors
Source: Sci Rep. 2021 Apr 29;11:9344. doi: 10.1038/s41598-021-88659-w (PMC8085177; doi:10.1038/s41598-021-88659-w)
Supplement: Supplementary file 1 — Supplementary Information. [file 41598_2021_88659_MOESM1_ESM.pdf]

A non-destructive sugar-feeding assay for parasite detection and estimating the extrinsic incubation period of *Plasmodium falciparum* in individual mosquito vectors

Authors: Edwige Guissou*[1,2,3,4], Jessica L. Waite*[5,6], Matthew Jones[5], Andrew S. Bell[5], Eunho Suh[5], Koudraogo B. Yameogo[1], Nicaise Djègbè[1], Dari F. Da[1], Domonbabele FdS Hien[1,3], Rakiswende S.Yerbanga[1,3], Anicet G. Ouedraogo[4], Kounbobr Roch Dabiré[1,3], Anna Cohuet[2,3], Matthew B. Thomas[5,7] and Thierry Lefèvre[1,2,3,8]

Affiliations
[1] Institut de Recherche en Sciences de la Santé, Bobo-Dioulasso, Burkina Faso.

[2] MIVEGEC, Montpellier University, IRD, CNRS, Montpellier, France.

[3] Laboratoire mixte international sur les vecteurs (LAMIVECT), Bobo Dioulasso, Burkina Faso.

[4] Université Nazi Boni, Bobo Dioulasso, Burkina Faso.

[5] Center for Infectious Disease Dynamics, The Pennsylvania State University, University Park, PA 16802, USA

[6] Green Mountain Antibodies, Inc. 1 Mill St. Suites 1-7, Burlington VT 05401 USA

[7] York Environmental Sustainability Institute and Department of Biology, University of York, UK.

[8] Centre de Recherche en Écologie et Évolution de la Santé (CREES), Montpellier, France.

*co-lead authors
Correspondence: edwigeguissou@yahoo.fr

Supplementary information files

Supplementary information file 1: Text S1. Assay development and optimization. Fig. S1.1. Experimental design. Table S1.1. Number of infected according to sample size and mosquito density. Fig. S1.2. Proportion of sporozoite infection according to sample type. Different letters (a, b, c) indicate differences between infection status. Table S1.2. Number of infected according to sample type. Fig. S1.3. Proportion of sporozoite infection according to sample type and parasite DNA extraction type. Table S1.3. Comparing effects of sample collection method and sample exposure time on sporozoite detection. Table S1.4. Evidence that our probe-based qPCR assay is functional, that sporozoites are deposited on sugar soaked filter paper squares, and that the assay works across mosquito species. Fig. S1.4. qPCR for 12 samples from cups of mosquitoes with good sporozoite infection (high prevalence and intensity). Table S1.5. Bass Assay vs. SYBR assay comparison. Table S1.6. Samples: sporozoite standard mix from infectious *An. stephensi*, quantified 135,000 spz/mL using hemocytometer. 40 glands in 1.2 mL PBS, portioned as follows into either an Eppendorf tube (liquid samples), or onto 15 mg cotton treated either with sugar solution, or left dry “plain”. Samples were either frozen immediately “frozen” or left in the 27 °C, 80 % rH conditions for 24 hrs prior to extraction and qPCR SYBR assay.

Supplementary information file 2: Text S2. Testing the assay at various temperatures. Table S2.1. Comparing likelihood of sporozoite detection using this assay at various temperatures.

Supplementary information file 3: Text S3. Oocyst rupturing and release of sporozoites. Fig. S3.1. Immature developing oocysts. Fig. S3.2. Mature and immature oocysts. Fig. S3.3. Ruptured and unruptured mature oocysts. Fig. S3.4. Ruptured oocysts.

Supplementary information file 4: Text S4. Infection level and mosquito survival in experiment 1. (a) Oocyst prevalence (± 95 % CI) on day 8-9 post-blood meal (dpbm). (b) Oocyst density at 8-9 dpbm. (c) Sporozoite prevalence (± 95 % CI) at 14-16 dpbm. (d) Sporozoite density at 14-16 dpbm. (e) Survival of mosquito females used to collect saliva for each parasite isolates.

Supplementary information file 5: Text S5. Infection level and mosquito survival in experiment 2. Fig. S5.1a. Infection load in mosquito females used to collect saliva. Fig. S5.1b. Estimated probability of *Plasmodium falciparum* carriage in cottons as a function of infection load in females used depositing saliva on these cottons. Fig. S5.2. Survival of mosquito females used to collect saliva for each anopheline species.

Supplementary information file 6: Text S6. Relationship between mosquito sugar feeding and *P. falciparum* positivity in cottons. Table S1: Evaluation of the presence colored fecal dots from 14 to 24 days after the infectious blood meal (dpbm) from 16 females infected with *P. falciparum*.

Supplementary information file 1: Assay development and optimization

Pilot experiments were conducted to determine the optimal substrate, extraction method and manipulation of substrate, assay duration, and minimum mosquito number in the sugar-feeding assay. Various detection methods using qPCR were also tested.

METHODS

<u>1. Optimizing mosquito sugar feeding substrate, manipulations, and mosquito density</u>

Methods IRSS: Laboratory-reared *An. gambiae* (Burkina) females were infected with two parasite isolates. At 7 days post blood meal, oocysts of some mosquito were examined to determine infection prevalence. At this stage, mosquitoes were placed individually or as groups of 2 or 3 placed in plastic drosophila tubes (diameter of 25 mm and height of 95 mm) covered on the top with a mosquito net (Fig. S1-1) to collect mosquito saliva. Cotton balls and whatman papers soaked with 10% glucose solution were deposited on the top of the tubes for mosquitoes feeding. Several exposure times of cotton balls and whatman papers (~1cm x 0.5cm) for mosquito were used: 2 hours, 13 hours, 24 hours and 48 hours.

Following this exposition period, the substrates were placed individually in sterile 1.5 mL tubes and stored at -20 °C for further processing. The presence of sporozoite in cotton balls, in whatman filter paper and in the mosquitoes used to collect saliva (carcasses) was determined using one of three DNA extraction methods: CTAB, DNAzol and Qiagen Dneasy Blood and Tissue Kits.

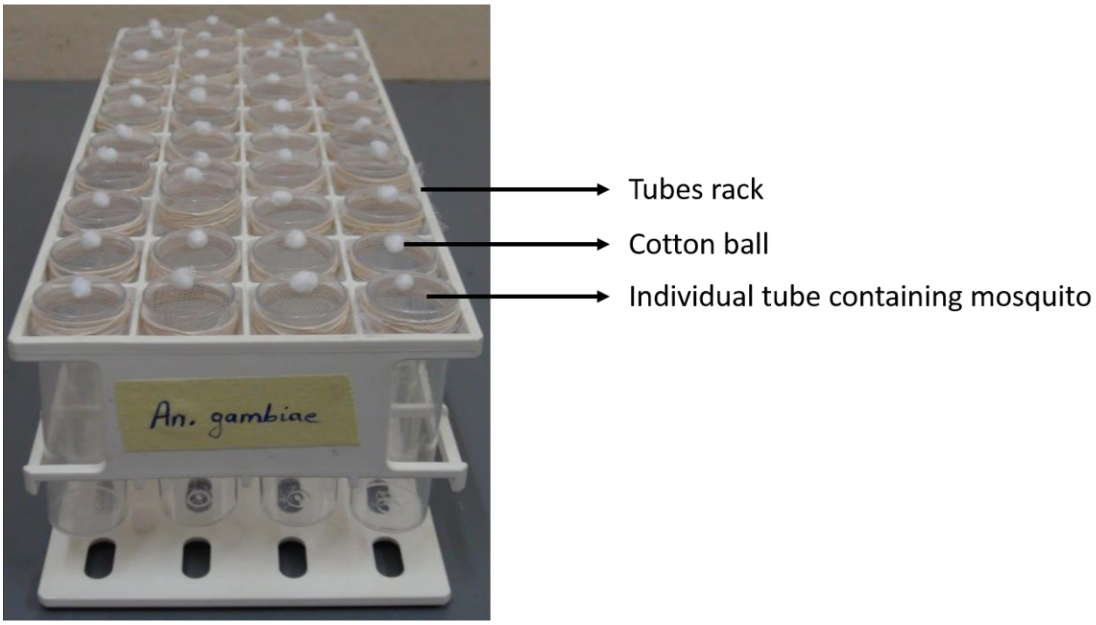

Fig. S1.1: Experimental design

Methods PSU: Mosquitoes (colony G3, a mix of the *An. gambiae* and *An. coluzzi* species versus *An. stephensi*) were housed in six 480 mL wax-lined paper cups total in groups of 120-150 and fed an infectious blood meal from *P. falciparum* culture, with 4 cups housing *An. stephensi* and 2 housing colony G3 mosquitoes. Cups were labelled according to mosquito species, and half of each set of cups was fed either dilution 1 (D1) or dilution 2 (D2) of the parasite culture. Dilution 1 had a higher concentration of parasite culture that resulted in higher prevalence and intensity of infection overall in mosquitoes compared to D2. All mosquitoes

were maintained at 27 °C until day 16 post infection with both a large sugar soaked cotton pad with PABA. Mosquitoes were removed for dissection daily to provide oocyst intensity and prevalence and sporozoite prevalence data. On days 15 and 16 post infection, the approximately 20-50 remaining groups of mosquitoes in these 6 cups were provided a small 1 cm x 0.5 cm Whatman filter paper rectangle (~1cm x 0.5cm) with a 15 mg piece of 10% glucose sugar soaked cotton wool on top of the filter paper to wet it for sampling sporozoites added in addition to a larger sugar soaked cotton wool used for maintenance. Filter papers with cottons were left on the netting on the top of each cup for a 24 hour period and then was collected into -20 °C. Only the filter paper was used for DNA extraction by the Qiagen kit method, cotton discarded assuming mosquitoes could not probe through the paper. Results are described in the results section 3: **Alternative qPCR probe-based method and other qPCR comparisons** since these samples were analyzed using an alternative probe-based qPCR method.

Note (PSU): Other substrates were also tested, but sugar soaked cotton resulted in the best feeding success and lowest mosquito mortality, and was easiest to manipulate. Liquid assays were tried with uninfected *An. stephensi* mosquitoes based on [1], with either open caps filled with dyed sugar solution on the bottom of the cup, or sugar solution in tubes covered with thin parafilm made accessible from the top of the cup. *Anopheles* mosquitoes preferred to sugar feed from the top of the cage rather than on the bottom from a liquid pool. Moreover, extraction of a leftover blood provided in a meal from a membrane feeder to infectious mosquitoes also resulted in a sporozoite positive DNA sample, but was messy and labor intensive, and feeding compliance daily may be low (although many mosquitoes will blood feed daily if provided the opportunity). Other sugar meal substrates including grape gelatin cubes on or off of filter paper on top of the cage netting, or sugar cubes or pieces of sugar cubes either did allow mosquitoes to feed well, left sticky reside, molded, or couldn't be easily collected (pers. obs. JLW, data not shown).

2. DNA extraction methods comparison

Extraction methods tested included CTAB, DNAzol extraction, and extraction using a Qiagen DNeasy Blood and Tissue kit. For the Qiagen kit we also tested how long to leave the cotton in the tube during the extraction, and found the best results when it was carried through to the end of the extraction, with AE elution buffer placed on the cotton and spin at the last step.

CTAB extraction: Parasite DNA from cottons and Whatman filter papers was extracted with Cetyl Trimethyl Ammonium Bromide 2% (CTAB 2%). Each sample was ground in 200 µl of a 2% solution of CTAB, which allows the inactivation of cellular nucleases. Cellular lysis was accentuated by placing the crushed mosquitoes for 5 min in a water bath at 65 °C. Then, 200 µl of chloroform was added. Samples were mixed by inversion and then centrifuged for 5 minutes at 12,000 rotations per min (rpm). The supernatant was removed into another 1.5 mL Eppendorf tube. In the tubes containing the supernatant, 200 µl of isopropanol was added and samples were mixed by inversion. These samples were centrifuged for 15 min at 12,000 rpm. After centrifugation, the supernatant iso-propanol mixture was emptied. Then, 200 µl of 70% ethanol was added and tubes were centrifuged for 5 min at 12,000 rpm. After this, 70% ethanol was emptied from the tube and the pellet dried for 5 min maximum at speed-vac. Finally, the DNA was recovered in 20 µl of ultrapure water and left in suspension on the bench all night before being stored at -20°C [2]. It was the same procedure for the extraction of parasite DNA from mosquito carcasses (head/thoraces).

DNAzol extraction: The samples of cotton and Whatman cards stored in 200 µl of DNAzol were ground. They were mixed by inversion and allowed to stand at room temperature for 5-10 min. At the end of this time, the samples were centrifuged at 14,000 rpm for 10 min. The supernatant was then transferred to a new 1.5 mL Eppendorf tube. The DNA was then precipitated by adding 200 µl of 100% ice-cold ethanol. The samples were mixed by inversion and incubated at -20 °C for 3 hours. Centrifugation of 14,000 rpm for 10 min was performed and the ethanol was removed. The pellet thus obtained was washed first with 75% ethanol at 14,000 rpm for 5 min and second with 75% ethanol at 7,500 rpm for 5 min. The ethanol was then removed by inversion and the pellet dried at Speed-Vac for 10 minutes. The DNA was dissolved in 50 µl of ultrapure water for half a day at room temperature [3]. The same procedure was used for the extraction of parasite DNA from mosquito carcasses (head/thoraces).

Qiagen DNeasy Blood and Tissue kit extraction: 180 µl of ATL buffer are added in the 1.5 mL tubes containing the cotton balls or Whatman cards and then samples were crushed. 20 µl of proteinase K was added and then vortexed for 30 sec before being incubated at 56 °C for 10 minutes in a water bath for lysis. 200 µl of Buffer AL was first added to the samples and then 200 µl of absolute ethanol (100%). The samples were then vortexed for 30 sec. The mixture obtained was transferred (together with the cotton balls or Whatman filter paper) into the columns (DNeasy Mini Spin Column). The columns were centrifuged at 8,000 rpm for 1 min and then transferred to new Qiagen tubes. The previous tubes containing the flow-through liquid were discarded. 500 µl of AW1 buffer was added and the columns were then centrifuged at 8,000 rpm for 1 min for the first wash. The second set of tubes was then discarded and the columns transferred to a third set of Qiagen tubes. 500 µl of AW2 buffer was then added before centrifuging the columns at 14,000 rpm for 3 minutes for the second wash. The third set of tubes was then discarded and the columns were then gently removed so that they would not come into contact with the liquid contained in the tube. The columns were transferred into Eppendorf 1.5 mL sterile tubes. The DNA contained in the column was solubilized in 50 µl of AE buffer and the tubes were then centrifuged at 8,000 rpm for 1 minute. The columns were then removed and eluted liquid contained in the 1.5 mL sterile Eppendorf tubes was stored at -20 °C for subsequent PCR. It was the same procedure for the extraction of parasite DNA from mosquito carcasses (head/thoraces).

Extraction methods using the Qiagen DNeasy kit at PSU were identical with the following minor exceptions. Samples were incubated at 56 °C on a shaker table heat block for microcentrifuge tubes set at 1,200 rpm for at least 10 minutes rather than in a Bain-Marie for lysis. Samples were additionally vortexed for 30s with the addition of Buffer AL. DNA samples were stored at -80°C for later PCR rather than -20°C.

Additionally, in Pennsylvania, the Qiagen DNeasy kit was used to compare the extraction efficiency when the cotton substrate was removed at different time points during the extraction procedure.

3. Alternative qPCR probe-based method and other qPCR comparisons

In addition to the qPCR methods described in the main manuscript, additional primer and amplification methods were tested. None performed as well as the SYBR qPCR methods described in the main manuscript, this probe based method also worked, but with a higher detection threshold.

3A: Probe-based qPCR

Parasites were quantified by quantitative real-time PCR by amplifying the cytochrome b gene of the *P. falciparum* parasite. 10 µl reaction conditions consisted of: 5 µl master mix, 1.2 µl forward primer, 1.2 µl reverse primer, 1.6 µl water, and 1 µl template DNA from the extracted product per reaction. The PCR kit PerfeCTa qPCR FastMix, UNG, Low ROX, Cat #95078 from Quanta BioSciences (VWR) was used. The following primer sequences were developed and used: forward primer qPCR-PfF (TTA CAT CAG GAA TGT TAT TGC), reverse primer qPCR-PfR (ATA TTG GAT CTC CTG CAA AT). Final primer concentrations were 240 nM. Samples were run with a minimum of a water negative control and positive control of *P. falciparum* DNA derived from 2 µl of *P. falciparum* in tissue culture extracted with a substrate to mimic initial trials for mosquito sugar feeding. Sporozoites were quantified using a 7500 Fast Real-time PCR System instrument, with thermocycler conditions set for 20 seconds of activation at 95°C, denaturation for 3 seconds at 95°C, and 30 seconds at 60°C to anneal and extend the product for 40 cycles.

3B: Bass assay and SYBR assay qPCR comparison
We compared the SYBR assay we used most consistently as described in the main methods section in the manuscript (under qPCR methods for *P. falciparum*) to the probe-based Bass assay described in [4] for detection of *P. falciparum*. A standard curve was created using 100 µl samples of a serially diluted sample of PBS spiked with *P. falciparum* NF54 strain culture placed onto 15mg cotton followed by extraction and comparative qPCR assays. Neat refers to the undiluted sample, -1 is diluted 1:10, -2 is diluted 1:100, -3 is diluted 1:1000, -4 is diluted 1:10,000. The unknowns tested in this assay are from sugar-soaked 15mg cotton substrate fed on overnight by *P. falciparum* infected *An. stephensi* mosquitoes on the 13th day post infection.

<u>4. Generating a standard curve and testing how sample handling processes might affect sample loss or degradation:</u>
Standard curve generation
Sporozoite infected from salivary glands of 40 infected mosquitoes and collected into 1,200 µl of PBS, and homogenized. The number of sporozoites was quantified using a hemocytometer. 100µl of this liquid solution was used to generate a standard curve by extraction using the Qiagen DNeasy kit and eluted into 50 µl AE buffer (as all cotton samples were treated in the main experiment). The extracted DNA of the infected salivary gland homogenate was serially diluted 1:2 a total of 8 times (1:1 through 1:128) to generate dilutions for use in a standard curve. These were run in triplicate. Additionally 1 and 2 µl of extracted DNA template was used to compare the effect of DNA template concentration.

Effects of heat, time, and processing on degradation
The salivary gland homogenate was also used to test sample handling and measure degradation or loss in the sampling process. Different amounts (5 ul, 20 ul, or 100 ul) of PBS-sporozoite mixture were pipetted either a) as liquid into a new 1.5 mL Eppendorf tube b) onto 15 mg dry cotton, c) onto cotton that had been dipped in sugar and left overnight in 27°C to simulate the moist cotton sample normally collected in this assay. The samples in the b and c treatments were duplicated, and half were placed directly in the freezer, and half were left in for 24hrs at 27 °C 80% rH before being capped and frozen the next day. The 100 µl sample was noticeably oversaturated compared to typical cottons used in the assay, but moisture in the 5µl and 20µl samples seemed within the normal moisture range of experimental cotton collected. All samples were extracted in the same extraction round and qPCR'd the following day. qPCR samples were run in duplicate.

RESULTS

1. Optimizing mosquito sugar feeding substrate, manipulations, and mosquito density

Results IRSS:

Mosquito number for assay

Mosquitoes housed in groups of 3 appear to give better sporozoite detection results than those housed individually or in groups of 2. However, since the test also worked for mosquitoes kept individually, and since individual conditioning is the only way to obtain a reliable estimate of the EIP or other relevant characteristics of the mosquito's life cycle, we chose use mosquito single conditioning for future experiments.

Table S1.1: Number of infected according to sample size and mosquito density
The detection rate expressed the proportion of *P. falciparum*-positive cotton or Whatman filter paper detected in qPCR on the number of samples analyzed in qPCR (according to sample type).

**Mosquito density**	**Sample**	**Infected (number)**	**Total (number)**	**Detection rate from 8 to 41 dpbm (%)**	**Chi-test**
Individual	Cotton	73	213	34	$X^2_1 = 5.41$; P = 0.02
	Whatman filter paper	40	171	23	
2	Cotton	19	53	36	$X^2_1 = 2.02$; P = 0.15
	Whatman filter paper	11	48	23	
3	Cotton	31	61	51	$X^2_1 = 2.70$; P = 0.10
	Whatman filter paper	22	61	36	

Optimal substrate

IRSS: The sporozoites detection was higher in cotton balls compared to Whatman filter paper. **(Table S1.1&S1.2&S1.3, Figs S1.2&S1.3).** Sporozoites was successfully detected in 123 of 328 cotton balls samples (37.5%) and in 73 of 280 Whatman filter papers samples (26%), (X^2_2 = 84.8; P = 0.002; Fig. 1; Table S2). The detection rate in cottons and in papers was low compared to the infection rate of sporozoites in females (57/57 = 100%) used to collect these cottons balls and whatman papers (X^2_2 = 96.7; P<0.0001; Fig. S1.2; Table S1.2).

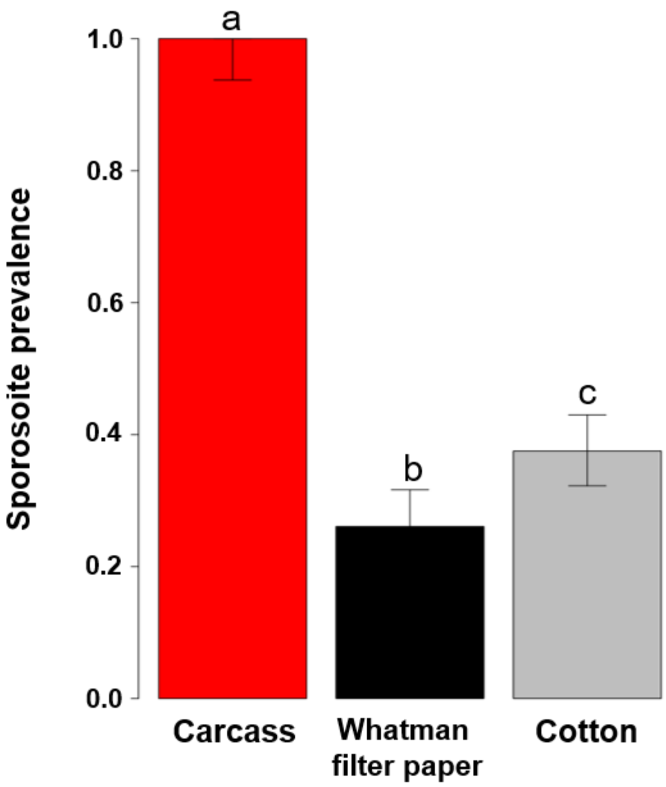

Fig. S1.2: Proportion of sporozoite infection according to sample type. Different letters (a, b, c) indicate differences between infection status. Red bar: carcasses of females used to collect saliva, black bar: whatman filter paper and gray bar: cotton.

Table S1.2: Number of infected according to sample type

Sample	Infected	Non infected	Infected Proportion	Chi-test
Cotton	123	205	37%	X^2_2 = 9.03; P<0.002
Whatman filter paper	73	207	26%	
Carcass	57	57	100%	X^2_2 = 109.89; P<0.0001

2. Optimal extraction method

The Qiagen extraction technique gives a better sporozoite detection rate than CTAB and DNAzol. In addition, for all three extraction techniques, mosquitoes produced more positive cotton balls (Qiagen extraction) than positive Whatman filter paper (X^2_1 = 15.36; P<0.0001; Fig.S1.3).

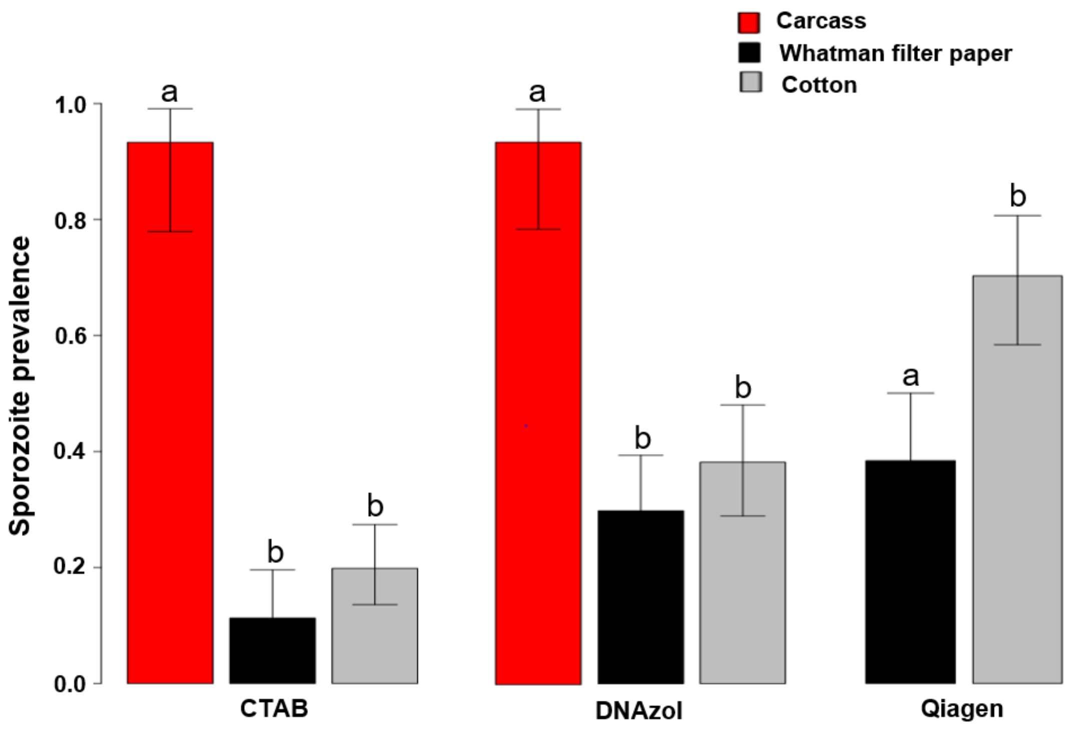

Fig. S1.3: Proportion of sporozoite detection according to sample type and parasite DNA extraction methods. Different letters (a, b) indicate differences in parasite detection between substrates compared within an extraction treatment. Red bar: carcasses of females used to collect saliva, black bar: whatman filter paper and gray bar: cotton. Note: no Qiagen-extracted carcasses

Exposure time / duration of assay

To compare exposure time, samples were divided into two groups. Exposure time in the first group was set at ≤ 13 hrs and included both cotton samples placed on gauze of infected mosquito housing for 13 hrs and Whatman filter papers similarly placed for 2 hrs. Exposure time in the second group was set to ≥ 24 hrs to increase sample size (24 and 48 hrs). More samples were detected positive using the exposure time of ≤ 13. Within this group, the more positive samples were detected using cotton compared to papers (X^2_1 = 10.68; P = 0.001, Table S3).

Table S1.3: Comparing effects of sample collection method and sample exposure time on sporozoite detection

Exposition time (hour)	Sample	Infected (number)	Total (number)	Infection proportion (%)	Chi-test
≤ 13	Cotton	95	230	41	X^2_1 = 10,68; P = 0,001
	Whatman filter paper	52	198	26	
≥ 24	Cotton	28	98	28	X^2_1 = 19,77; P = 0,66
	Whatman filter paper	21	82	26	

Increasing the exposure time of cotton balls or Whatman filter paper did not improve sporozoite detection. A single exposure period (13 hours) was sufficient.

3. Alternative qPCR probe-based method and other qPCR comparisons

3A: Probe based qPCR assay: Ct values from MJ (Matthew Jones) method of primer/probe qPCRs (lower than 35 cycles is considered positive), paired with sporozoite prevalence data from dissections. Ordering of sample identification (ID) is researcher, dilution (D1 is more concentrated than D2), Anopheline mosquito species, and number of days post infectious feed on which the sample of filter paper with sugar-soaked cotton on top was collected. There were 6 cups used (4 *An. stephensi,* 2 *An. gambiae/coluzzi* G3 strain) of around 20-50 mosquitoes for the previous 24 hours as described in methods section S.1B. Only the filter paper was further processed (small sugared cottons on top of filter papers were discarded).

Table S1.4. Evidence that our probe-based qPCR assay is functional, that sporozoites are deposited on sugar soaked filter paper squares, and that the assay works across mosquito species.

Sample ID	Ct value	Oocyst prevalence and (intensity) 8dpbm by dissection	Spz prev. (closest dpbm)
ES, D1, stephensi, 15 dpbm	36.224	100% (37.1)	100% (17)
ES, D2, stephensi, 15 dpbm	Undetermined	100% (32.7) *ave of days 7&9	90% (17)
JLW, D1, stephensi, 15 dpbm	36.4231	100% (24.2)	100% (17)
JLW, D2, stephensi, 15 dpbm	36.3595	100% (21.0)	100% (12), 80% (17)
ES, D1, gambiae 15 dpbm	30.562	100% (46.9)	100% (17)
ES, D2, gambiae 15 dpbm	Undetermined	100% (18.1)	100% (12), 92% (17)
ES, D1, stephensi, 16 dpbm	37.038	100% (37.1)	100% (17)
ES, D2, stephensi, 16 dpbm	Undetermined	100% (32.7) *ave of days 7&9	90% (17)
JLW, D1, stephensi, 16 dpbm	33.2054	100% (24.2)	100% (17)
JLW, D2, stephensi, 16 dpbm	33.3272	100% (21.0)	100% (12), 80% (17)
ES, D1, gambiae 16 dpbm	35.1362	100% (46.9)	100% (17)
ES, D2, gambiae 16 dpbm	33.8087	100% (18.1)	100% (12), 92% (17)
NTC, water – negative control	Undetermined	NA	NA
Positive control – #7 2 µl culture	26.2676	NA	NA

3B: Bass assay and SYBR assay qPCR comparison:

Compared to our SYBR method described in the main text, the Bass Assay probe-based qPCR did not perform quite as well by generating more false negatives compared to SYBR and having lower sensitivity (See Table S1.5).

Table S1.5 Bass Assay vs. SYBR assay comparison: Ct represents the density of sporozoites in the different samples. NF54 is a strain of *P. falciparum* (*P.f.*).

**Sample Info**	**Sample #ID**	**Bass Assay Ct**	**SYBR Assay Ct**
100 µl Plain cotton spiked with 1:10 PBS to NF54 culture, diluted	Neat	28.0818	20.8941
	Neat	28.1586	20.6531
	-1	31.5902	23.6893
	-1	31.3893	23.7328
	-2	35.4549	27.076
	-2	36.1595	27.049
	-3	38.2602	30.3946
	-3	Undetermined	**31.3999**
	-4	Undetermined	Undetermined
	-4	Undetermined	Undetermined
cotton	NTC	Undetermined	Undetermined
cotton	NTC	Undetermined	Undetermined
An. stephensi infected with NF54 *P.f*, 13 dpbm cottons	As-8 d13	35.0596	27.1948
	As-8 d13	36.0864	27.0398
	As-10 d13	37.6146	30.4648
	As-10 d13	Undetermined	**30.0864**
	As-12 d13	36.1339	29.1585
	As-12 d13	37.0737	29.009

Assay	Slope	Intercept	R2
Bass	-3.559072	42.388142	0.986662
SYBR	-3.372236	34.041691	0.991813

While both assays detected parasites, the SYBR assay is roughly 100x more sensitive and also resulted in a better overall efficiency. Although the Bass Assay was not further optimized in our lab, it seemed that the SYBR assay performed better.

4. Generating a standard curve and testing how sample handling processes might affect sample loss or degradation:

Standard curve generation

The infected salivary glands homogenate from the dissection of 40 *An. stephensi* mosquitoes diluted in 1.2 mL 1 x PBS was estimated to contain 135,000 sporozoites per mL based on calculations scaling up from the number of sporozoites visualized in the hemocytometer counts to the volume of liquid used in the hemocytometer counting. The number of extracted sporozoites then used for the neat sample extraction for generating the standard curve is roughly 270 sporozoites per µl (in 50 µl total). This SYBR assay targets the COX1 mitochondrial gene, and each sporozoite has 20 copies of this gene [5], thus there are 5,400 copies of the gene in 1µl of the undiluted sample from this extraction. It was calculated that in 100 µl of this salivary gland homogenate that there would be 13,500 sporozoites, in 20 µl 2700 sporozoites, and in 5 µl 675 sporozoites (all multiplied by 20 for expected gene copy numbers).

Serial dilutions 1:2 a total of 8 times (1:1 through 1:128) used in the standard curve showed the expected results of a slightly better efficiency with 2 µl template samples. Overall results showed that increased template volume resulted in lower Ct values, as expected. It was determined from these data that the minimum detection threshold was lower than the 1:128 dilution, for which the Ct value was 26.11-27.62 depending on whether 1 µl or 2 µl of template was used (lower values 2 ul). Thus, it is estimated that the assay can detect <675 sporozoites. This makes sense, given that mosquitoes are thought to expectorate in the order of 10's to 100's of sporozoites at one feeding, and likely on the lower end of this range for sugar feeding, see [5,6] and [7].

Effects of heat, time, and processing on degradation

The effect of 24 hours at 27 °C was quite strong with most samples left at 27 °C overnight performing worse than their counterparts that were frozen immediately after their addition to either sugar soaked or dry cottons. Liquid only samples should not have had any trouble with DNA loss attributed to sample being stuck on the cotton, and indeed this was the case with most liquid samples performing the best of their set for template volume (lowest Ct). Averages are presented in Table S1.6 of samples run in duplicate. In bold are samples that weren't as close as is preferable for qPCR values since pipetting 1µl accurately can be challenging, and at low parasite numbers, the likelihood of picking up DNA from low concentrations and getting it into a reaction could be stochastic.

From these results, it can be observed that larger amounts of sporozoites added to the assay (mostly) resulted in lower Ct values as expected. There was evidence that heat contributed to sample degradation with both varieties of cotton (either treated with a sugar solution prior to use, or left dry and plain before sporozoite homogenate was added) had lower Ct scores on average when immediately frozen compared to after 24 hours in the warm and humid conditions. Liquid had lower Ct scores for every volume, suggesting that there is some loss with parasite DNA sticking to the cotton or inefficiencies in the extraction even after optimization, as would be expected. However, this loss was not severe, resulting in only a slightly lower Ct score in most comparisons.

Table S1.6: Samples: sporozoite standard mix from infectious *An. stephensi*, quantified 135,000 spz/mL using hemocytometer. 40 glands in 1.2 mls PBS, portioned as follows into either an Eppendorf tube (liquid samples), or onto 15 mg cotton treated either with sugar solution, or left dry "plain". Samples were either frozen immediately "frozen" or left in the 27 °C, 80% rH conditions for 24 hrs prior to extraction and qPCR SYBR assay.

Sample volume used	Cotton with sugar	Plain cotton	Liquid (frozen)	24hr Cotton w/ sugar	24hr Plain cotton
5ul	27.1804	28.91635	25.8291	27.8003	30.3973
20ul	26.3881	27.2387	24.35025	**32.99555**	29.70025
100ul	23.2631	21.5206	21.3335	29.204	29.33915

References cited

1. Ye, Y. H. *et al.* Evolutionary potential of the extrinsic incubation period of dengue virus in *Aedes aegypti*. *Evolution (N. Y).* 2459–2469 (2016). doi:10.1111/evo.13039
2. Doyle, J. . & Doyle, J. . DNA Isolation from Small Amount of Plant Tissue. *Phytochem. Bull.* **57**, (1991).
3. Ausubel, F. M. *et al. Current Protocols in Molecular Biology*. *Molecular Biology* (2003). doi:10.1002/mrd.1080010210
4. Melanson, V. R. *et al.* Improving vector-borne pathogen surveillance: A laboratory-based study exploring the potential to detect dengue virus and malaria parasites in mosquito saliva. *J. Vector Borne Dis.* (2017). doi:10.4103/0972-9062.225834
5. Marie, A. *et al.* Evaluation of a real-time quantitative PCR to measure the wild *Plasmodium falciparum* infectivity rate in salivary glands of *Anopheles gambiae*. *Malar. J.* **12**, 224 (2013).
6. Churcher, T. S. *et al.* Probability of Transmission of Malaria from Mosquito to Human Is Regulated by Mosquito Parasite Density in Naïve and Vaccinated Hosts. *PLoS Pathog.* (2017). doi:10.1371/journal.ppat.1006108
7. Frischknecht, F. *et al.* Imaging movement of malaria parasites during transmission by *Anopheles* mosquitoes. *Cell. Microbiol.* **6**, (2004).

Supplementary information file 2: Testing the assay at various temperatures

Methods

With the goal of testing whether it was possible to use the optimized assay at various temperatures, two additional experiments were conducted.

To test lower thermal limits, 9 cups holding 6-12 *An. stephensi* mosquitoes were maintained at 27 °C for 15 days following an infectious feed then were placed in the following temperatures: (3 x 12 °C, 2 x 14 °C, 2 x 16 °C, and 2 x 18 °C). The standard optimized sugar soaked cotton assay was run from 16-21 dpbm with cotton collected daily from each cup, with at least one mosquito surviving in each cup during this sampling period. Samples were analyzed by Qiagen extraction and qPCR using the best SYBR methods described in the main paper. Additionally, individual *An. stephensi* mosquitoes maintained at 27 °C for 25 days post infection at which time 12 were placed at 32 °C and 12 were placed at 34 °C. All mosquitoes in both experiments were estimated to have infection prevalence of over 90% with *P. falciparum* from infectious feeds on cultured parasites. Mosquitoes in the warmer temperatures were sampled at 26 and 27 dpbm. It was expected that sugar feeding at warmer temperatures would be more frequent compared to lower temperatures to maintain hydration. In lower temperatures mosquitoes would also have reduced activity levels, and, as shown from sugar feeding assays described in the main text, lower temperatures also reduce potentially sugar feeding and/or digestion rate.

Results

Table S2. Comparing likelihood of sporozoite detection using this assay at various temperatures.

**Temperature °C**	**Total sample number**	**Sporozoite positive samples (dpbm)**	**% Positive samples**
12 °C	18	0	0 %
14 °C	12	2 (16, 21)	16.67 %
16 °C	12	1 (17)	8.3 %
18 °C	12	6 (17, 19, 20, 21)	50 %
32 °C	22	7 (26, 27)	31.8 %
34 °C	24	3 (26)	12.5 %

These results show that mosquitoes kept at temperatures <16°C and >32°C were less likely to take a sugar compared to those kept between 18°C-32°C. This suggests that the use of the spit assay would be more optimal at a temperature between 18°C and 32°C and this will avoid false negative sample.

Supplementary information file 3: Oocyst rupture assay

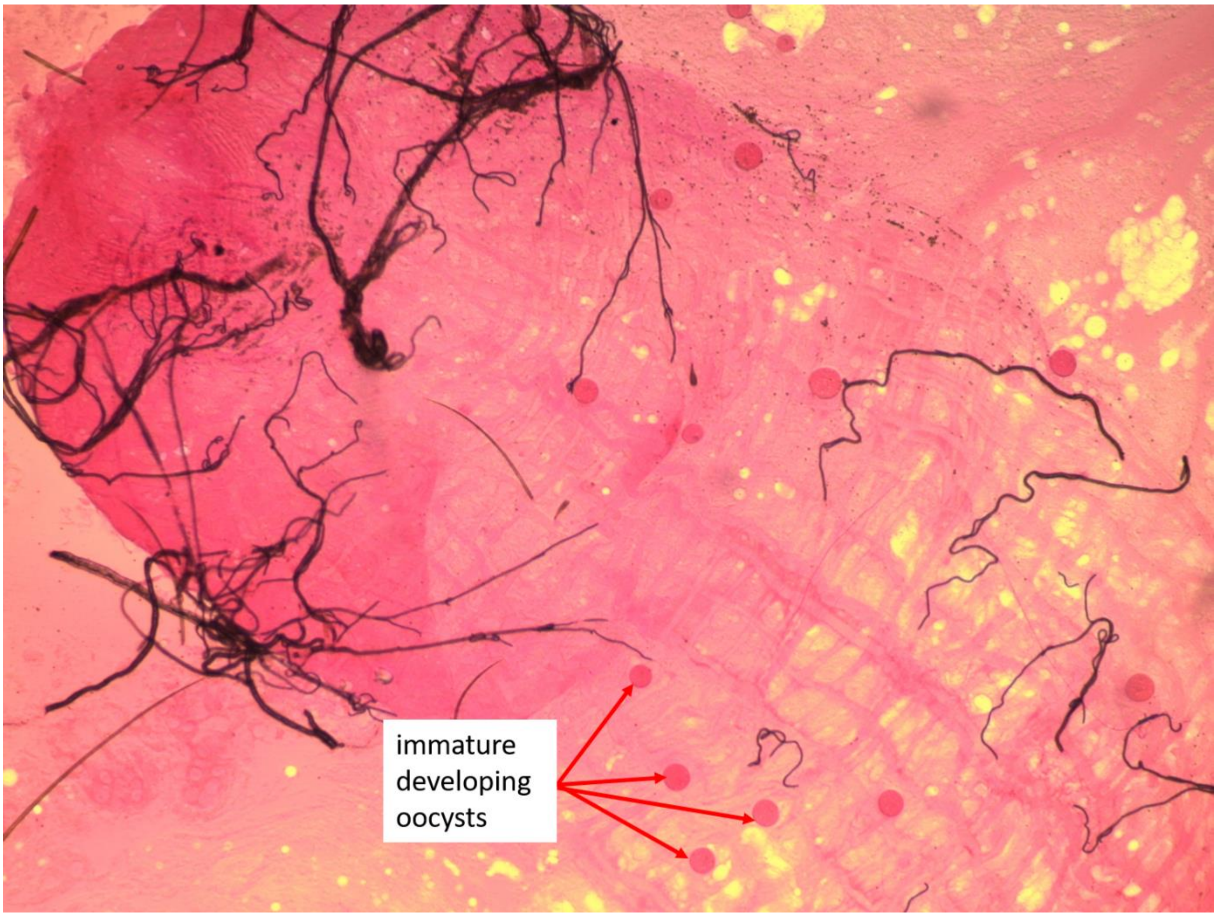

Fig. S3-1: Microscopic observation (x10) of a mosquito gut dissected at 6 days post-blood-meal (dpbm) and harboring a dozen of immature developing oocysts. Mosquitoes were maintained at 27°C during parasite development. Credit: Guissou Edwige

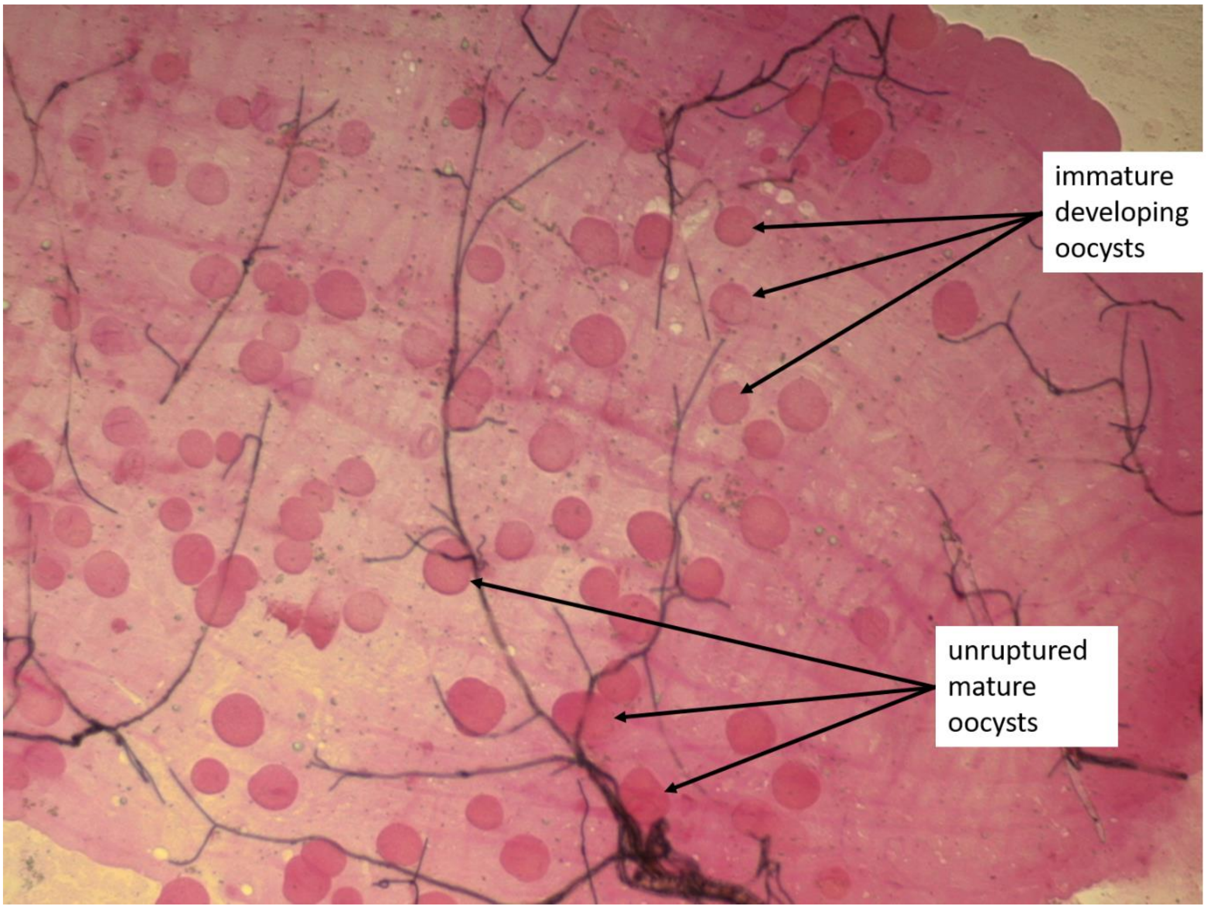

Fig. S3-2: Microscopic observation (x 10) of a mosquito gut dissected at 8 days post-blood-meal (dpbm) and harboring both mature and immature oocysts. Note the protrusion of the capsule of some oocysts that may here result from the application of the coverslip. In our experiment, these distorted oocysts were recorded as intact unruptured oocysts because the capsule has not broken yet. Mosquitoes were maintained at 27°C during parasite development. Credit: Guissou Edwige

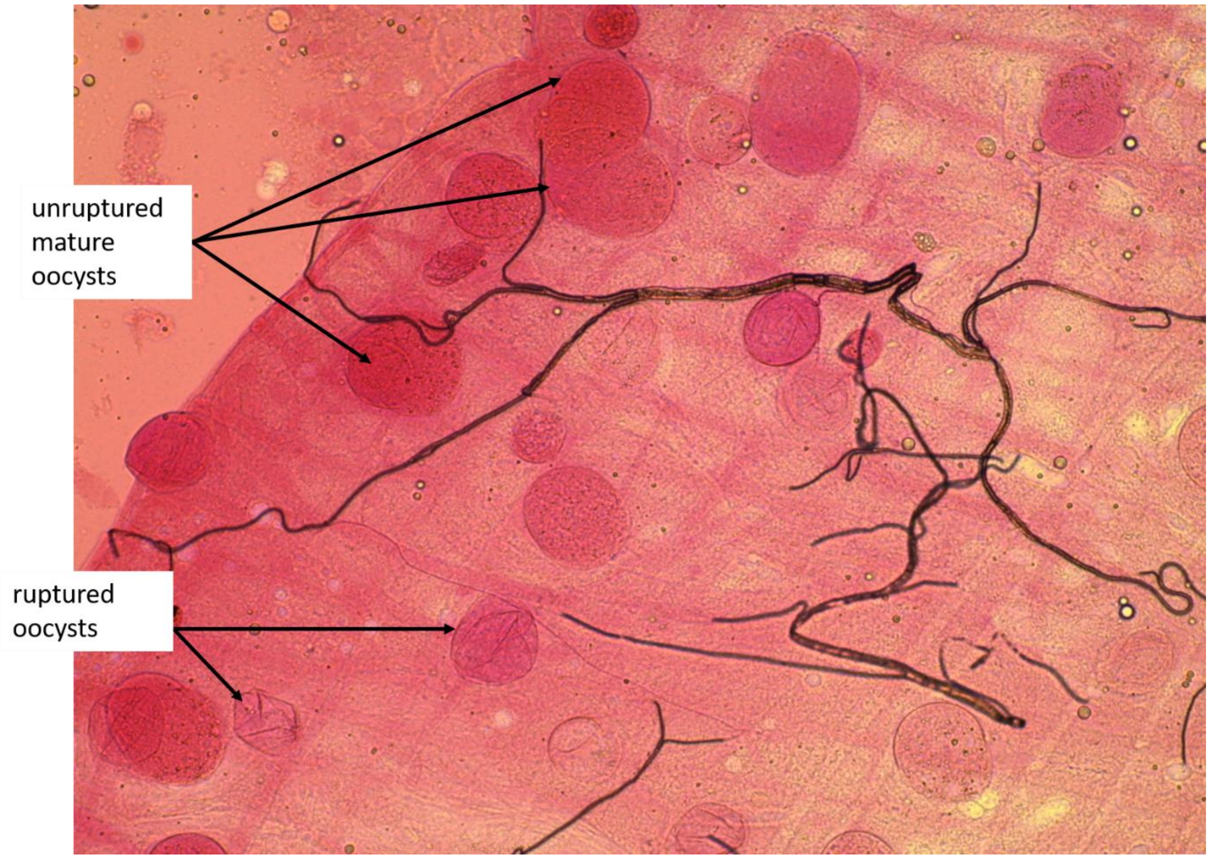

Fig. S3-3: Microscopic observation (x 20) of a mosquito gut dissected at 12 days post-blood-meal (dpbm) and harboring both ruptured and unruptured mature oocysts. The ruptured oocysts are characterized by empty and withered capsules. Ruptured oocysts look like a hatched egg from which only the shell remains. Mosquitoes were maintained at 27°C during parasite development. Credit: Guissou Edwige

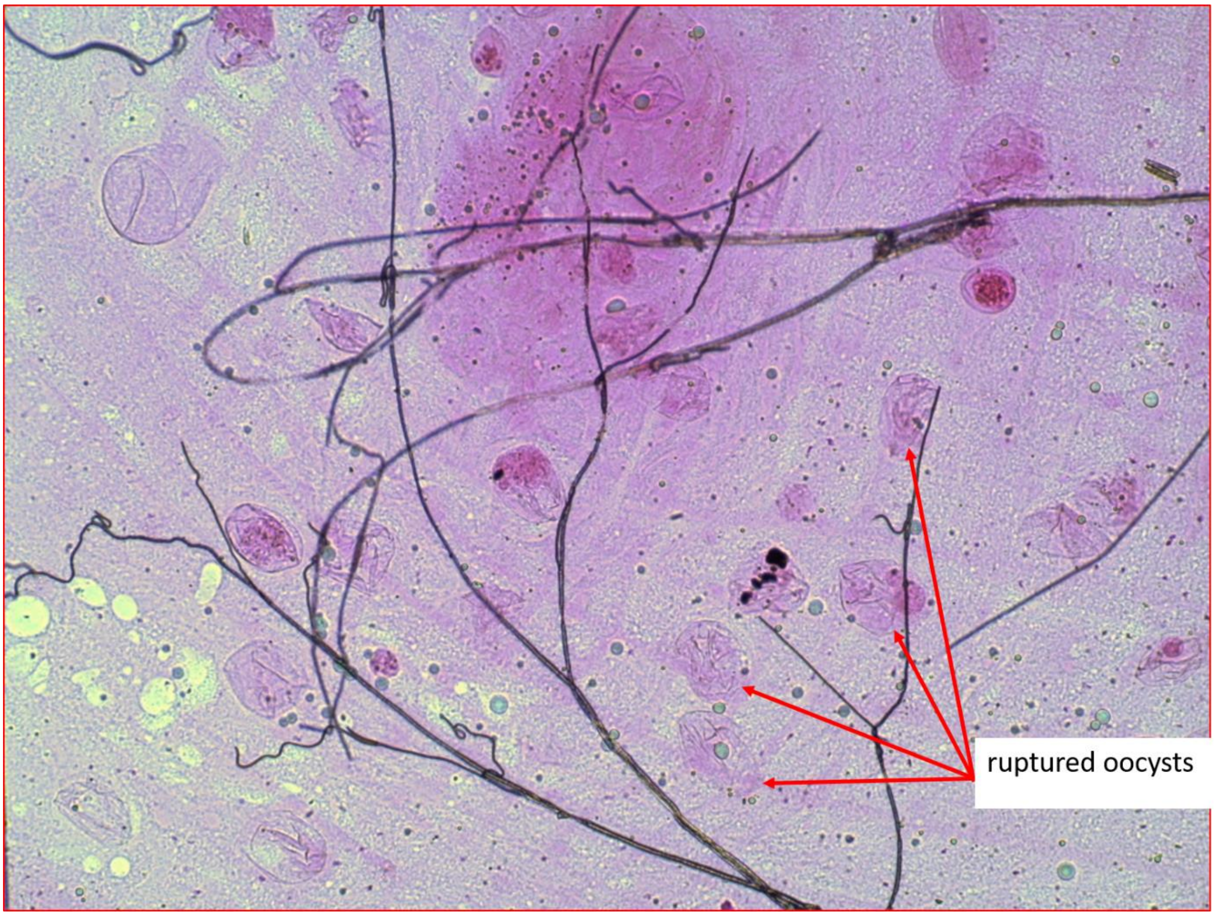

Fig. S3-4: Microscopic observation (x 20) of a mosquito gut dissected at 12 days post-blood-meal (dpbm) and harboring ruptured oocysts only. The ruptured oocysts are characterized by empty and withered capsules. Ruptured oocysts look like a hatched egg from which only the shell remains. Mosquitoes were maintained at 27°C during parasite development. Credit: Guissou Edwige

Supplementary information file 4

1. Infection level and mosquito survivorship in experiment named "Comparing estimates of the parasite's EIP using the classic dissection approach versus the non-destructive individual "spit" assay")

The proportion of infected mosquitoes (prevalence) in isolate A (95 % (20/22)) and in isolate B (100 % (20/20)) was statistically similar (GLM binomial: $LRT\ X^2_1 = 1.31$, P = 0.25, Fig. S4a). The mean number (± se) of developing oocysts in infected females (density) was significantly higher for isolate B (191.65 ± 21) than for A (13.86 ± 2) (GLM negative binomial: $LRT\ X^2_1 = 133$, P < 0.001, Fig. S4b). In addition, the mean number (± se) of ruptured oocysts in infected females (density) was significantly higher for isolate B (71.19 ± 6.86) than for A (12.57 ± 1.25) (GLM negative binomial: $LRT\ X^2_1 = 1658$, P < 0.001, Fig. S4c). Although the prevalence of sporozoites based on microscopic observation from 14 to 16 dpbm was similar for both parasite isolates (A: 95 % (37/39); B: 97 % (36/37); $LRT\ X^2_1 = 0.30$, P = 0.58, Fig. S4d left panel), that based on qPCR was slightly higher for isolate B (100 % (37/37)), than for A (92 % (36/39) ($LRT\ X^2_1 = 4.12$, P = 0.04, Fig. S4d). The sporozoites density based on the scores was higher for isolate B (median = 3) than for A (median = 2), ($LRT\ X^2_1 = 1.65$, P < 0.001, Fig. S4e), thus confirming the oocyst observation. However, the sporozoite density based on qPCR (Ct) was similar for both parasite isolates ($LRT\ X^2_1 = 0.004$, P = 0.53, Fig. S4e). Mosquito survival was not significantly associated to parasite isolates (survival cox model: (LRT $X^2_1 = 0.54$, P = 0.46. Fig. S4f).

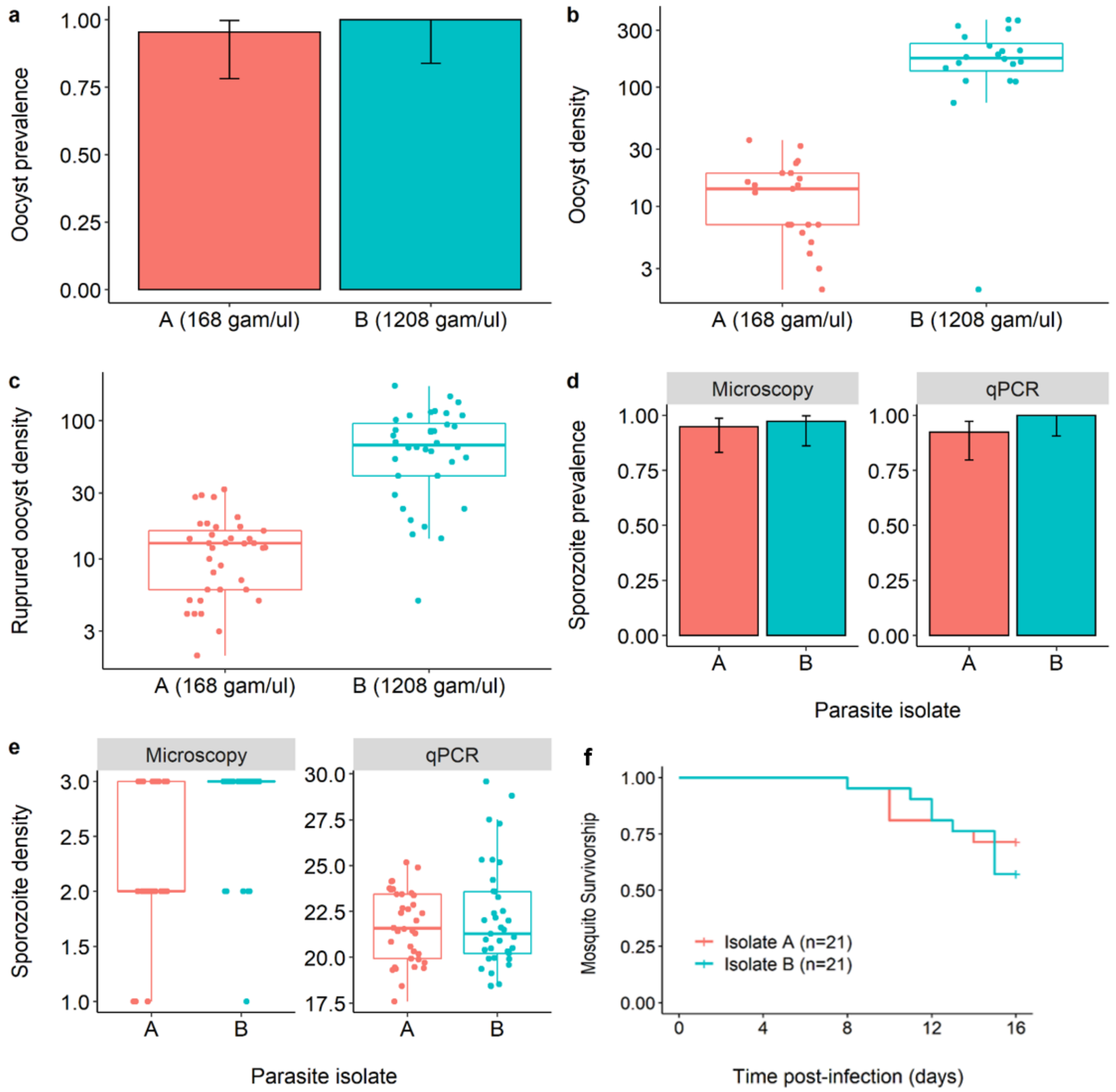

Fig. S4.1: Infection level at two distinct time points over the course of infection (oocyst and sporozoite stages). a: Oocyst prevalence (± 95% CI) on day 8-9 post-blood meal (dpbm), expressed as the number of mosquito females harboring at least one oocyst in their midguts out of the total number of dissected females, for each parasite isolate (red bar: isolate A, blue bar: isolate B). b: Oocyst density at 8-9 dpbm, expressed as the mean number of developing oocysts (± se) in the guts of infected females, for each parasite isolate. The different numbers in parentheses in Fig. 1a and 1b indicate gametocymia (number of gametocytes / µl of blood) for isolates A and B. c: Ruptured oocyst density at 14-16 dpbm, expressed as the mean number of ruptured oocysts (± se) in the guts of infected females, for each parasite isolate. The different numbers in parentheses in Fig. 1c indicate mosquito number for each isolate. d: Sporozoite prevalence (± 95% CI) at 14-16 dpbm, expressed as the number of mosquito salivary glands detected positive to *P. falciparum* using microscopic observation (left panel) or qPCR (right panel) out of the total number of dissected salivary glands, for each parasite isolates. e: Sporozoite density at 14-16 dpbm, expressed as the median score assigned to each salivary gland (1: a few sporozoites, 2: a moderate level of sporozoites, 3: numerous sporozoites) when using microscopy for parasite detection or the median Ct values when using qPCR (the lower the Ct, the higher the sporozoite density) for each parasite isolate. f: Survival of mosquito females used to collect saliva for each parasite isolates.

2. Relationship between the proportion of ruptured oocysts and the total number of oocysts

One hundred and twenty-one female mosquitoes exposed to parasite A isolate and 114 to parasite B isolate were dissected at 8 to 16 dpbm to assess microscopically the presence and number of oocysts in the midguts and sporozoites in the salivary glands. At 8 and 9 dpbm, no ruptured oocyst were observed and all individuals dissected during these two first days were excluded from the analysis of the relationship between the proportion of ruptured oocysts and the total number of oocysts. The individual proportions of ruptured oocysts were arcsine square root transformed prior analysis. The effect of oocyst density on the transformed response variable was tested using a GLM with Gaussian errors. Out of 190 dissected individuals of 10-16 dpbm, we found that the proportion of ruptured oocysts increased when the total number of oocysts decreased (*LRT* $X^2_1 = 16$, $P < 0.0001$, Fig. S4.2).

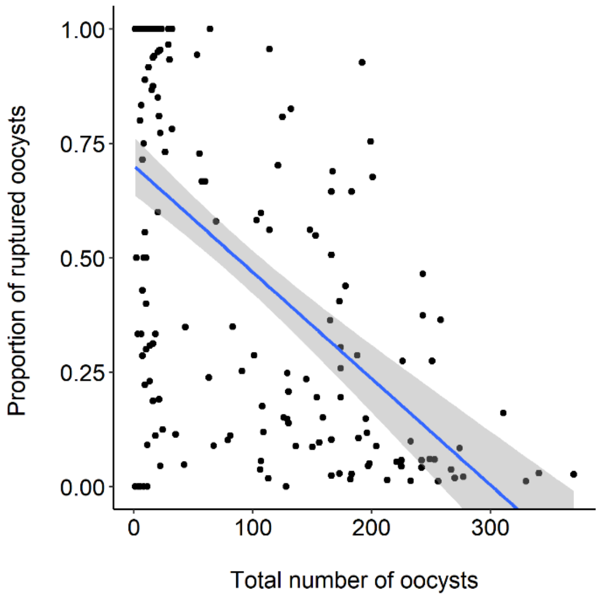

Fig. S4.2: The proportion of ruptured oocysts as a function of the total number of oocysts in individual mosquitoes. The proportion of ruptured oocysts is the number of ruptured oocysts out of the total number of oocysts in the midguts of infected mosquitoes from 10-16 dpbm. Each black dot represent a mosquito individual and the blue line represents the best-fit linear relationship (± se).

Supplementary information file 5: Infection level and mosquito survival in experiment named "Individual estimation of EIP in different mosquito species"

The parasite load (inferred from the Ct values: the higher the Ct, the lower the parasite load) in infected *An. coluzzii* carcasses was lower than that of *An. gambiae* and *An. arabiensis* (mean Ct ± se: 26.64 ± 1.24, 23.6 ± 0.86 and 21.51 ± 0.6, respectively, KW $X^2{}_2$ = 9.3, P = 0.009, Fig. S5.1a). In addition, there was a positive relationship between the probability to generate *P. falciparum* positive cotton samples and infection intensity in individual females (LRT $X^2{}_1$ = 10, P = 0.002, Fig. S5.1b).

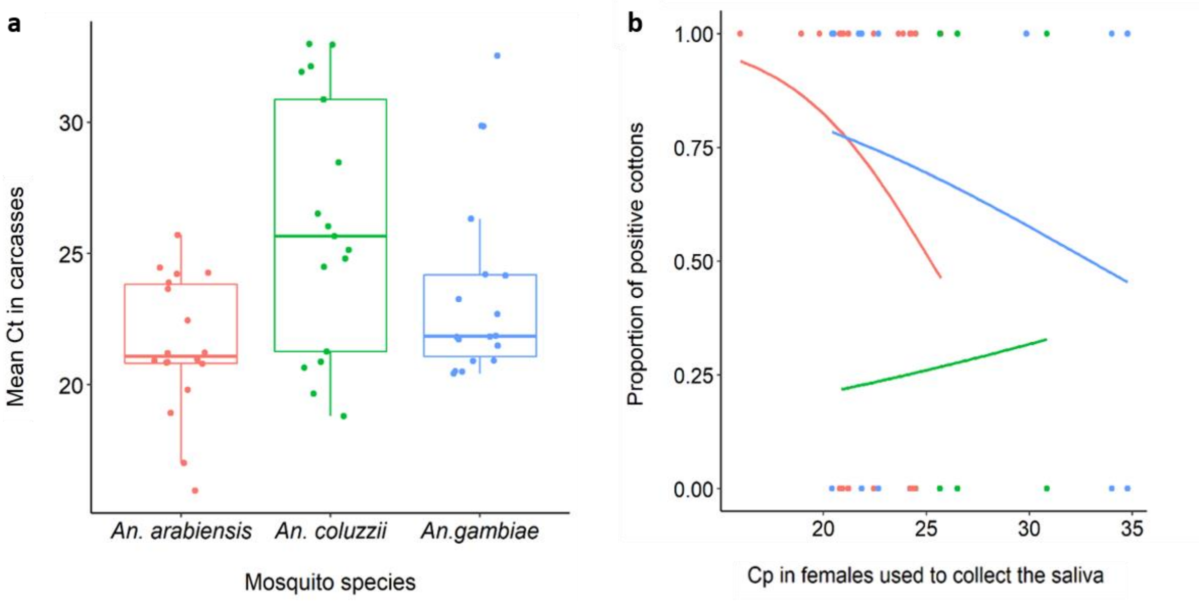

Fig.S5.1. Infection level. (a) Infection load in mosquito females used to collect saliva (expressed as the mean Ct values from the qPCR output. The lower the Ct, the higher the parasite load) for each anopheline species (red: *An. arabiensis, green: An. coluzzii,* blue: *An. gambiae).* (b) Estimated probability of *P. falciparum* carriage in cottons as a function of infection load in females used depositing saliva on these cottons. The lower the Ct, the higher the infection load. The red, blue and green lines show the relationship for *An. arabiensis, An. coluzzii* and *An. gambiae* respectively.

An. arabiensis survived better in plastic tubes than both *An. coluzzii* and *An. gambiae* (survival cox analysis on infected and uninfected individuals (n = 60): LRT $X^2{}_2$ = 7.8. P = 0.02, median survivorship: 18.5, 12 and 12.5 days, in *An. arabiensis*, *An. coluzzii* and *An. gambiae*, respectively, Fig. S5.2).

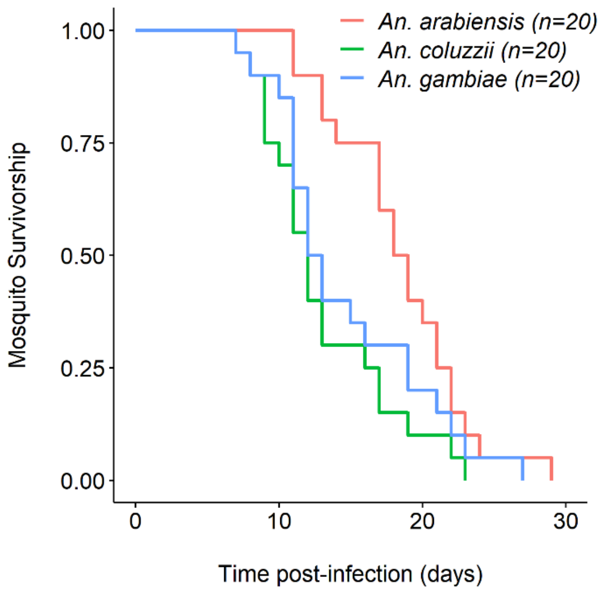

Fig S5.2. Survival of mosquito females used to collect saliva for each anopheline species.

Supplementary information file 6: Relationship between mosquito sugar feeding and *P. falciparum* positivity in cottons

Table S6: Evaluation of the presence colored fecal dots from 14 to 24 days after the infectious blood meal (dpbm) from 16 females infected with *P. falciparum*. Each id represents a female. Color_dpbm represents the day of cotton collection and the observation (1) or not (0) of colored fecal dots. The death column corresponds to the day the mosquito died. Red color: *P. falciparum* positive sample, Blue color: *P. falciparum* negative sample, NA: not available sample

**Id**	**color_dpbm14**	**color_dpbm15**	**color_dpbm16**	**color_dpbm17**	**color_dpbm18**	**color_dpbm19**	**color_dpbm20**	**color_dpbm21**	**color_dpbm22**	**color_dpbm23**	**color_dpbm24**	**Death(dpbm)**
2	0	0	1	1	NA	NA	NA	NA	NA	NA	NA	18
4	0	0	1	0	1	0	0	NA	NA	NA	NA	21
6	1	0	0	NA	NA	NA	NA	NA	NA	NA	NA	16
7	1	1	1	NA	NA	NA	NA	NA	NA	NA	NA	17
10	1	1	1	1	1	NA	NA	NA	NA	NA	NA	19
11	1	1	1	1	NA	NA	NA	NA	NA	NA	NA	18
13	1	1	1	1	1	1	1	1	1	1	1	24
15	0	1	1	1	1	0	1	1	1	1	NA	23
16	NA	0	0	1	1	NA	NA	NA	NA	NA	NA	18
18	0	0	1	1	1	1	0	NA	NA	NA	NA	21
19	1	1	1	1	1	1	0	NA	NA	NA	NA	21
20	1	1	1	1	0	0	NA	NA	NA	NA	NA	20
21	0	0	0	0	1	0	0	0	0	0	1	24
23	0	1	1	NA	NA	NA	NA	NA	NA	NA	NA	17
26	1	1	NA	NA	NA	NA	NA	NA	NA	NA	NA	15
30	1	1	1	NA	NA	NA	NA	NA	NA	NA	NA	16

This table shows that the probability of detecting *P. falciparum* positive cottons was not related to the detection of colored fecal dots on the papers. We must therefore continue the investigations in order to know the frequency of sugar feeding intake of mosquitoes because this will improve the efficiency of the spit assay.
